# Two chromium(II) acetate complexes with N-heterocyclic carbene (NHC) coligands

**DOI:** 10.1107/S2056989024005796

**Published:** 2024-06-28

**Authors:** Christian Heiser, Kurt Merzweiler

**Affiliations:** a Martin-Luther-Universität Halle, Naturwissenschaftliche Fakultät II, Institut für Chemie, Germany; Universidad Nacional Autónoma de México, México

**Keywords:** crystal structure, chromium, acetate, NHC, paddle-wheel

## Abstract

Tetra­kis(μ-acetato-κ^2^*O*:*O*′)bis­{[1,3-bis­(2,6-diiso­propyl­phen­yl)imidazol-2-yl­idene-κ*C*^2^]chromium(II)} tetra­hydro­furan disolvate, [Cr_2_(C_2_H_3_O_2_)_4_(C_27_H_36_N_4_)_2_]·2C_4_H_8_O or [Cr_2_(OAc)_4_(IDipp)_2_]·2C_4_H_8_O (**1**), and tetra­kis­(μ-acetato-κ^2^*O*:*O*′)bis­{[1,3-bis­(2,4,6-tri­methyl­phen­yl)imidazol-2-yl­idene-κ*C*^2^]chromium(II)},{Cr_2_(C_2_H_3_O_2_)_4_(C_21_H_24_N_2_)_2_] or [Cr_2_(OAc)_4_(IMes)_2_] (**2**), were synthesized from anhydrous chromium(II) acetate [Cr_2_(OAc)_4_] and the corresponding NHC (NHC = N-heterocyclic carbene) in toluene as solvent. Both complexes crystallize in the triclinic system, space group *P*. The mol­ecular structures consist of Cr_2_(OAc)_4_ paddle-wheels that carry two terminal NHC ligands. This leads to a square-pyramidal coordination of the chromium atoms.

## Abstract 2

The crystal structures of two chromium(II) acetate complexes with N-heterocyclic carbene coligands were determined.

## 1. Chemical context

Since its discovery in 1844 by Peligot (Peligot *et al.*, 1844 ▸), chromium(II) acetate has frequently been used as the starting material for a large variety of chromium(II) compounds (Cotton *et al.*, 2005 ▸). Treatment of chromium(II) acetate with donor ligands *L* gives dinuclear complexes [Cr_2_(OAc)_4_*L*_2_] that adopt paddle-wheel structures with the ligands *L* at axial positions. This structure pattern was first observed for the dihydrate [Cr_2_(OAc)_4_(H_2_O)_2_] (van Niekerk *et al.*, 1953 ▸; Cotton *et al.*, 1971 ▸) and later on for a large number of ligands comprising oxygen and, particularly, nitro­gen donor atoms (Cotton *et al.*, 2005 ▸).

Recently, we reported on chromium(II) silyl­amide complexes that were generated from chromium(II) acetate as starting material. In the course of these investigations, the application of NHC coligands proved very successful. Typically, a suspension of chromium(II) acetate in THF was first treated with the NHC ligand to give deeply violet-coloured solutions. Treatment of the *in situ* generated chromium(II) acetate NHC complex with Li_2_Me_2_Si(NPh)_2_ led to [Cr{Me_2_Si(NPh)_2_(NHC)_2_}] (Heiser & Merzweiler, 2022 ▸). We were now inter­ested in the isolation and structural characterization of chromium(II) acetate NHC complexes.

Several X-ray crystal structures of chromium(II) NHC complexes are reported in the literature. The first references date back to the late 1990s when the crystal structures of [Mes_2_Cr(IPr)_2_] [IPr = 1,3-bis­(diisoprop­yl)imidazol-2-yl­idene; Danopoulos *et al.*, 1997 ▸], [CpPhCr(IMes)] (Voges *et al.*, 1999 ▸) and [Cp_2_Cr(IMes)] (IMes = 1,3-bis­(2,4,6-tri­methyl­phen­yl)limidazol-2-yl­idene; Abernethy *et al.*, 1999 ▸) were published. Apart from organo chromium(II) compounds, some CrCl_2_ NHC complexes have been studied. Typical examples are [Cr_2_Cl_4_((IPrMe_2_))_2_(THF)_2_], [CrCl_2_(IPrMe_2_)_2_] (IPrMe_2_ = 1,3-diisopropyl-4,5-dimethyl-imidazol-2-yl­idene; Wang *et al.*, 2010 ▸) and [CrCl_2_(IDipp)_2_] [IDipp = 1,3-bis­(2,6-diiso­propyl­phen­yl)imidazol-2-yl­idene; Jones *et al.*, 2012 ▸]. Moreover, chelating bis­(NHC) ligands and NHC ligands with additional donor functionality have been applied in chromium(II) chemistry, *e.g.* CSD refcodes DERNUK (Kreisel *et al.*, 2006 ▸), QIBKUI (Kreisel *et al.* 2007 ▸), BEKGAB (Conde-Guadano *et al.*, 2012*a* ▸), QUGFAB (Simler *et al.*, 2015 ▸), SAVNOW (Ashida *et al.*, 2022 ▸), SEDMEU (Pugh *et al.*, 2006 ▸) and ZEKCEZ (Conde-Guadano *et al.*, 2012*b* ▸).
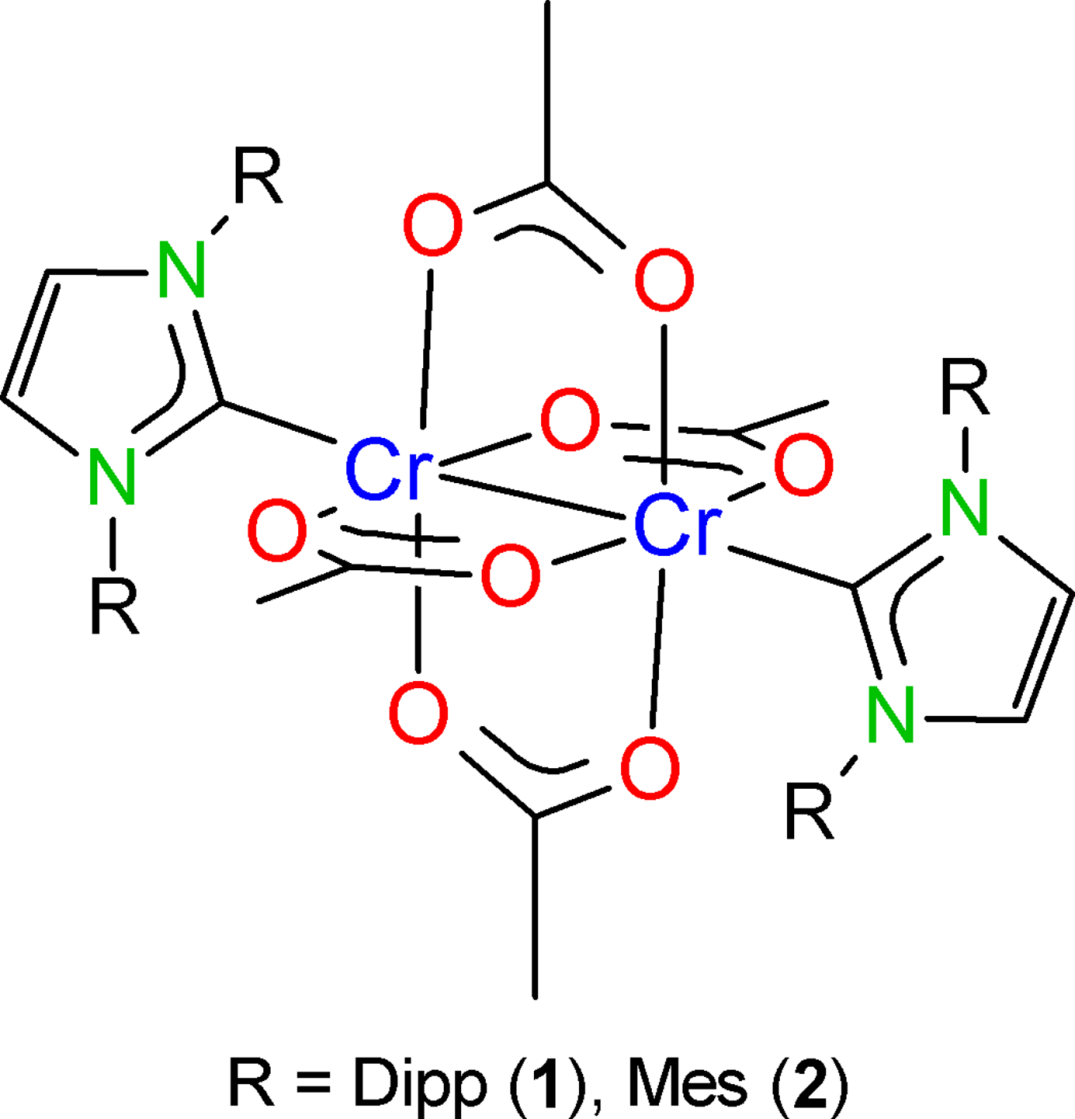


## 2. Structural commentary

**Figure 1:**
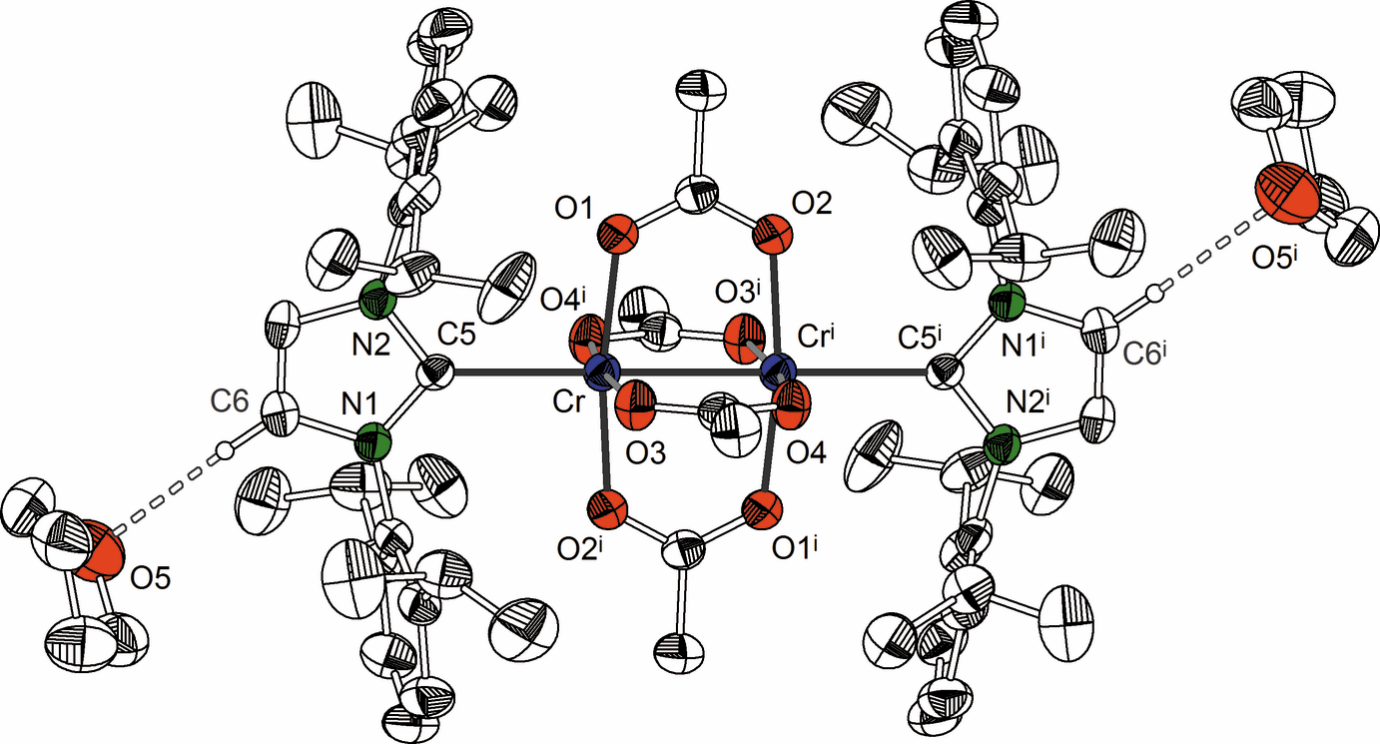
Mol­ecular structure of **1** in the crystal. Displacement ellipsoids are at the 50% probability level. There is a disorder over two orientations concerning the THF mol­ecule and three of the ^i^Pr groups. In each case, only the major orientation is displayed. H atoms not involved in C—H⋯O hydrogen bonds are omitted for clarity. Inter­molecular C—H⋯O hydrogen bonds shown as dashed lines. [Symmetry code: (i) −*x* + 1; −*y* + 1; −*z* + 1.

The title compounds (Figs. 1 ▸ and 2 ▸) were obtained by reacting anyhdrous chromium(II) acetate with the corresponding NHC in toluene. After filtration, the solutions were cooled to obtain [Cr_2_(OAc)_4_(IDipp)_2_]·2THF (**1**) and [Cr_2_(OAc)_4_(IMes)_2_] (**2**) in the form of violet crystals. Single crystals suitable for X-ray diffraction were obtained by recrystallization from THF (compound **1**) and toluene (compound **2**).

[Cr_2_(OAc)_4_(IDipp)_2_]·2THF (**1**) and [Cr_2_(OAc)_4_(IMes)_2_] (**2**) crystallize in the triclinic system, space group *P*
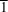
with *Z* = 1. The crystal structure of **1** consists of discrete [Cr_2_(OAc)_4_(IDipp)_2_] units and two mol­ecules of tetra­hydro­furan per formula unit. Compound **2** crystallizes without solvate mol­ecules. In both complexes, the Cr_2_(OAc)_4_ units exhibit classical paddle-wheel structures with crystallographically imposed 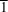 symmetry. The coordination sphere of the chromium atoms consists of four acetate oxygen atoms at the base of a square pyramid and the NHC carbon atom at the apex.

The Cr—O distances in **1** range from 2.012 (2) to 2.025 (1) Å and in **2** from 2.024 (2) to 2.027 (2) Å (Tables 1 ▸ and 2 ▸). Similar distances have been reported for 15 chromium(II) acetate derivatives that are currently deposited in the CSD database (Groom *et al.*, 2016 ▸). The shortest Cr—O(acetate) distance [1.988 (5) Å] was observed in [Cr_2_(OAc)_4_] and the largest one [2.034 (1) Å] was found in [Cr_2_(OAc)_4_(*trans*-bie)_2_] [*trans*-bie = 2,2′-ethene-1,2-diylbis(1-methyl-1*H*-imidazole); Fritsch *et al.*, 2014 ▸].

The Cr—C(NHC) distances in **1** and **2** are 2.381 (2) and 2.365 (3) Å, respectively. These values are roughly comparable to the Cr—O and Cr—N distances in chromium(II) acetate complexes with axial O and N donor ligands. In the case of O donor ligands, the Cr—O distances vary from 2.257 to 2.306 Å. For N donor ligands, the range is 2.274–2.415 Å. In square-planar [Cr*R*_2_(NHC)_2_] complexes (*R* = organyl, halogen) the Cr—C bonds are markedly shorter compared to those in compounds **1** and **2**, *e.g.* [CrCl_2_(IDipp)_2_] [Cr—C: 2.148 (2)–2.162 (2) Å; Jones *et al.*, 2012 ▸] and [CrCl_2_(IPrMe)_2_] [Cr—C: 2.159 (3)–2.163 (2) Å; Jones *et al.*, 2012 ▸]. The shortest Cr—C distance (2.0930 Å) was observed for a NHC pincer ligand (CSD code QUGFAB; Simler *et al.*, 2015 ▸) and the largest (2.180 Å) for [CrPh_2_(IPrMe)_2_] (Wang *et al.*, 2011 ▸).

The Cr—Cr distances in compounds **1** [2.5308 (6) Å] and **2** [2.5284 (9) Å] are significantly larger than in comparable [Cr_2_(OAc)_4_*L*_2_] complexes with N and O donor ligands. According to the CSD database, the Cr—Cr distances vary from 2.270 to 2.452 Å with a median of 2.348 Å. The larger Cr—Cr distances in **1** and **2** also become apparent in a slight pyramidalization of the CrO_4_ units. The distances of the chromium atoms from the mean plane through the four O atoms are 0.1516 (3) Å for compound **1** and 0.1476 (4) Å in the case of compound **2**. Moreover, the C—Cr—O angles significantly exceed 90° [**1**: 92.78 (5)–95.67 (5)°, **2**: 93.88 (8)–94.46 (8)°].

Overall, the geometric parameters of both compounds are very similar. However, it is worth mentioning that complexes **1** and **2** differ in the mutual orientation of the NHC ligands and the paddle-wheel core. In the case of compound **1**, the imidazolidine ring adopts an eclipsed orientation with respect to the O1–Cr–O2 unit as indicated by the torsion angles N2—C5—Cr—O1 [1.2 (2)°] and N1—C5—Cr—O2^i^ [−7.5 (2)°]. By contrast, a staggered conformation is found in compound **2** with torsion angles of 48.3 (3)° (N1—C5—Cr—O1) and 45.7 (3)° (N2—C5—Cr—O2^i^). It is obvious to assume that the steric repulsion between the *iso*-propyl groups of the NHC ligand and acetate methyl groups prevents a staggered orientation of the imidazoline ring in compound **1**.

## 3. Supra­molecular features

**Figure 3:**
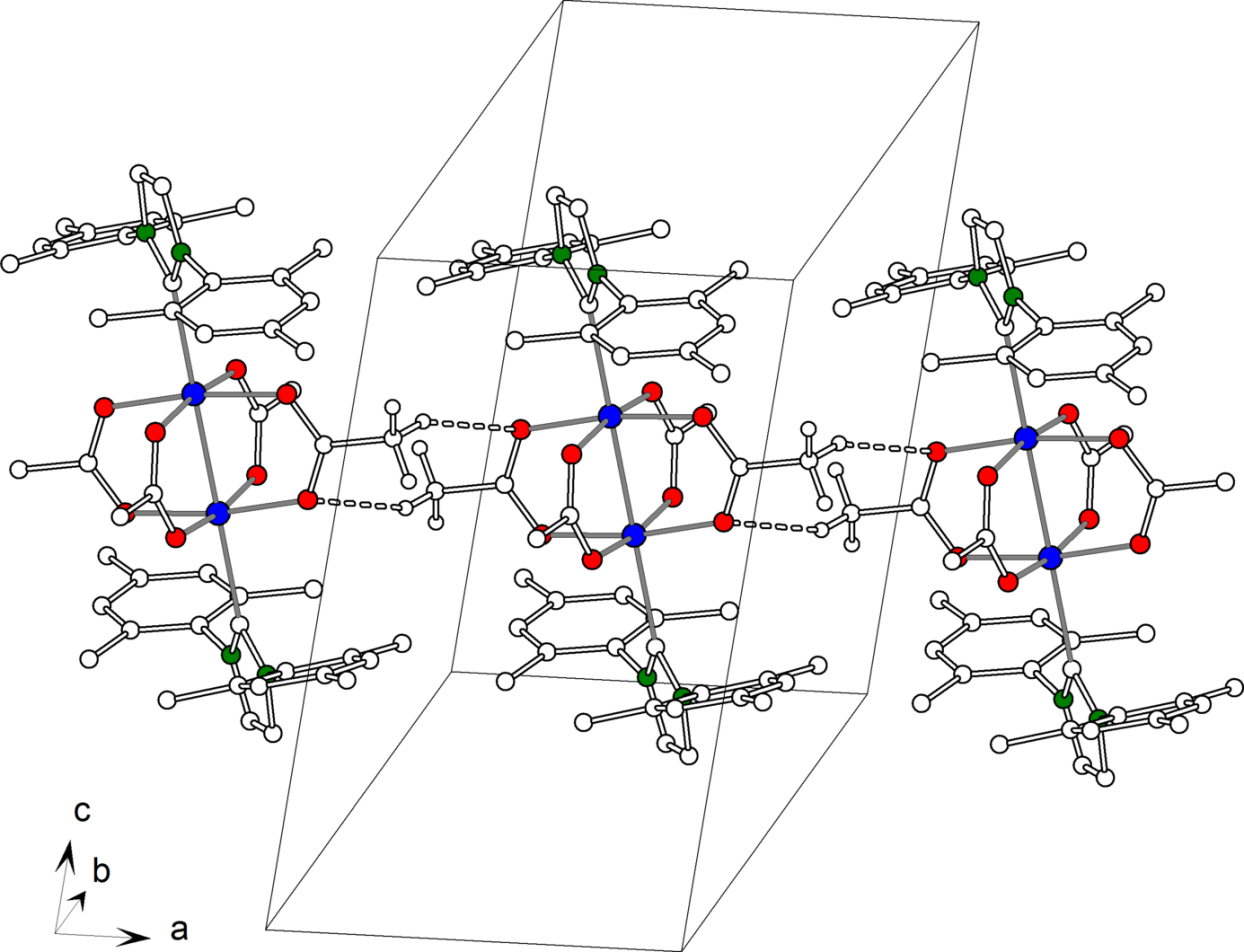
Crystal structure of **2**, inter­molecular C—H⋯O hydrogen bonds are shown as dashed lines.

**Figure 4:**
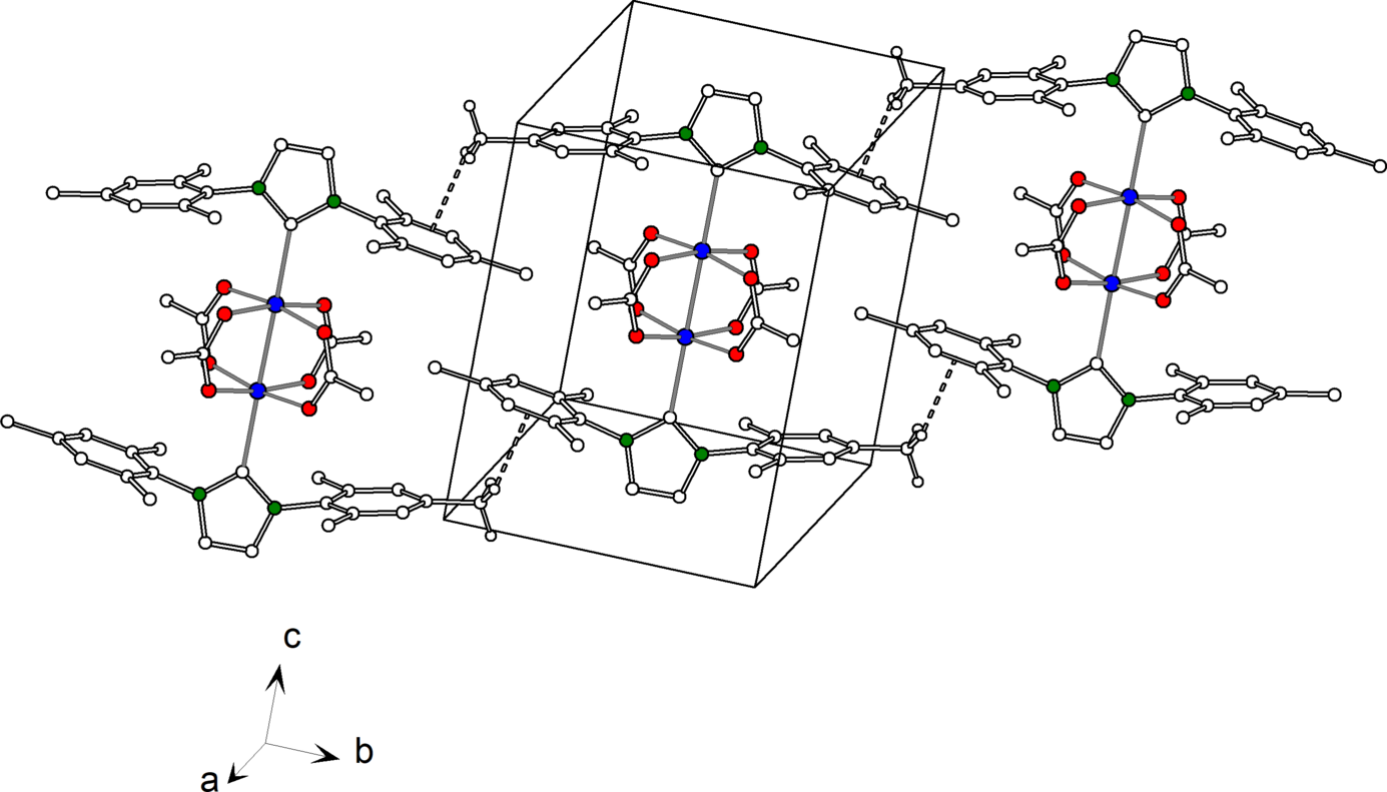
Crystal structure of **2**, inter­molecular C—H⋯π hydrogen bonds shown as dashed lines.

Compound **1** displays a weak C—H⋯O hydrogen bridge [*D*⋯*A*: 3.411 (6) Å; Table 3 ▸] between the C6—H6 group of the imidazolidine ring and the tetra­hydro­furan oxygen atom O5. In the case of compound **2**, there is a weak C—H⋯O hydrogen bridge [*D*⋯*A*: 3.527 (4) Å; Table 4 ▸, Fig. 3 ▸] between the acetate carbon atom C2 and the acetate oxygen atom O2^ii^ of a neighbouring complex unit. Furthermore, there is a complementary hydrogen bridge between C2^ii^ and O2. As a result, the chromium acetate complexes are catenated by 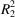
(8) hydrogen-bond motifs along the direction of the crystallographic a axis. Moreover, the supra­molecular structure is supported by weak C—H⋯π hydrogen bonds (Fig. 4 ▸), which are formed between neighbouring mesityl groups. The distance between the methyl carbon atom C15 and the centroid of the aromatic ring C17^iii^–C22^iii^ is 3.340 (4) Å.

## 4. Database survey

A search in the Cambridge Structural Database (CSD, Version 5.45, 2024; Groom *et al.*, 2016 ▸) revealed 21 crystal structures of chromium(II) acetate complexes, CSD refcodes: ACETCR (Cotton & Rice, 1978 ▸), ACPCRA (Cotton & Felthouse, 1980 ▸), ACPCRB (Cotton & Felthouse, 1980 ▸), ACPCRB01 (Huang *et al.*, 2018 ▸), CRAQAC (van Niekerk *et al.*, 1953 ▸), CRAQAC03 (Benard *et al.*, 1980 ▸), CRAQAC11 (Cotton *et al.*, 1971 ▸), CRAQAC12 (Benard *et al.*, 1980 ▸), CRAQAC13 (Herich *et al.*, 2018 ▸), CUCSEA (Fritsch *et al.*, 2014 ▸), CUYSEU (Cotton & Wang, 1984 ▸), CUYSIY (Cotton & Wang, 1984 ▸), KETXOZ (Huang *et al.*, 2018 ▸), KETXOZ01 (Huang *et al.*, 2018 ▸), KETYAM (Huang *et al.*, 2018 ▸), KETYAM01 (Huang *et al.*, 2018 ▸), KETYEQ (Huang *et al.*, 2018 ▸), KETYEQ01 (Huang *et al.*, 2018 ▸), LIRTAH (Cotton *et al.*, 2000 ▸), PIPACR (Cotton & Rice, 1978 ▸, XIYCER (Heiser & Merzweiler, 2023 ▸). Most of them contain N- and O-donor ligands. No NHC adducts of chromium(II) acetate have been reported so far.

## 5. Synthesis and crystallization

All manipulations were carried out under an argon atmosphere using standard Schlenk techniques. Toluene and THF were dried over sodium/benzo­phenone and freshly distilled prior to use. Chromium(II) acetate (Brauer, 1981 ▸), 1,3-bis­(2,6-diiso­propyl­phen­yl)imidazolidine-2-yl­idene (IDipp) and 1,3-bis­(1,3,5-tri­methyl­phen­yl)imidazolidine-2-yl­idene (IMes) (Medici *et al.*, 2018 ▸) were prepared according to literature methods.

**Synthesis of [Cr_2_(OAc)_4_(IDipp)_2_] (1)**

To a suspension of chromium(II) acetate (470 mg, 1.39 mmol) in toluene (12 ml) was added a solution of IDipp (1180 mg, 2.78 mmol) in toluene (8 ml). The solution was stirred at 300 K overnight. The chromium(II) acetate dissolved and a change of colour from dark red to violet was observed. Insoluble material was filtered off and the solution was concentrated slightly *in vacuo*. Upon standing at 248 K for two days, the product crystallized in the form of violet crystals, which were filtered off and dried *in vacuo*. Single crystals of the product were obtained upon cooling down a THF solution of **1** to 248 K. Yield: 660 mg (40%).

C_70_H_100_Cr_2_N_4_O_10_ (1261.53 g mol^−1^). C 66.8 (calc. 66.6); H 7.9 (calc. 7.6); N 5.0 (calc. 5.0) %.

IR (ATR): ν = 3135 *w*, 3075 *w*, 3021 *w*, 2963 *m*, 2929 *m*, 2870 *m*, 1608 *s*, 1574 *s*, 1537 *s*, 1496 *m*, 1435 *s*, 1389 *s*, 1330 *m*, 1304 *m*, 1259 *m*, 1209 *m*, 1182 *m*, 1150 *m*, 1103 *m*, 1060 *m*, 1042 *m*, 1030 *m*, 951 *m*, 937 *m*, 908 *m*, 868 *m*, 808 *s*, 801 *s*, 754 *s*, 735 *m*, 674 *s*, 621 *s*, 594 *m*, 540 *s*, 519 *m*, 466 *s*, 441 *s*, 416 *s* cm^−1^.

**Synthesis of [Cr_2_(OAc)_4_(IMes)_2_] (2)**

To a suspension of chromium(II) acetate (580 mg, 1.71 mmol) in toluene (10 ml) was added a solution of IMes (1050 mg, 3.41 mmol) in toluene (10 ml). The solution was stirred at room temperature for 30 minutes, and after that it was heated to 313 K for one h, during which time the chromium(II) acetate dissolved and the solution turned violet. The solution was filtered while hot and washed with hot toluene (2 × 5 ml). After reducing the volume to half the amount, the solution was heated to dissolve the precipitated product. Upon standing at 267 K for two days, the product crystallized in a form of violet single crystals, which were filtered off and dried *in vacuo*. Yield: 650 mg (40%).

C_50_H_60_Cr_2_N_4_O_8_ (949.02 g mol^−1^). C 63.3 (calc. 63.3); H 6.0 (6.4); N 5.9 (5.9) %.

IR (ATR): ν = 3128 *w*, 3005 *w*, 2976 *w*, 2915 *m*, 2858 *w*, 1606 *s*, 1539 *m*, 1485 *m*, 1424 *s*, 1390 *m*, 1337 *m*, 1287 *m*, 1254 *m*, 1230 *m*, 1210 *m*, 1157 *m*, 1085 *m*, 1065 *m*, 1036 *m*, 1022 *m*, 961 *m*, 926 *m*, 870 *w*, 841 *m*, 742 *w*, 733 *m*, 720 *m*, 673 *s*, 641 *m*, 619 *m*, 591 *m*, 574 *m*, 509 *m*, 497 *m*, 467 *m*, 448 *w*, 381 *vs*, 331 *m*, 307 *m*, 276 *s*, 253 *m*, 227 *s*, 209 *s* cm^−1^.

## 6. Refinement

Crystal data, data collection and structure refinement details are summarized in Table 5 ▸. All hydrogen atoms were positioned geometrically and refined using a riding model with *U*_iso_(H) = 1.2(CH and CH_2_) or 1.5(CH_3_) times *U*_eq_(C).

The THF mol­ecule in compound **1** is disordered over two positions with an occupation ratio of 0.795 (12)/0.205 (12)/. The isopropyl groups C17–C19, C26–C28 and C29–C31 are disordered over two positions with occupation ratios of 0.62 (4)/0.38 (4), 0.60 (4)/0.40 (4) and 0.81 (3)/0.19 (3), respectively.

## Supplementary Material

Crystal structure: contains datablock(s) 1, 2. DOI: 10.1107/S2056989024005796/jq2035sup1.cif

Structure factors: contains datablock(s) 1. DOI: 10.1107/S2056989024005796/jq20351sup2.hkl

Structure factors: contains datablock(s) 2. DOI: 10.1107/S2056989024005796/jq20352sup3.hkl

CCDC references: 2362906, 2362905

Additional supporting information:  crystallographic information; 3D view; checkCIF report

## Figures and Tables

**Figure 2:**
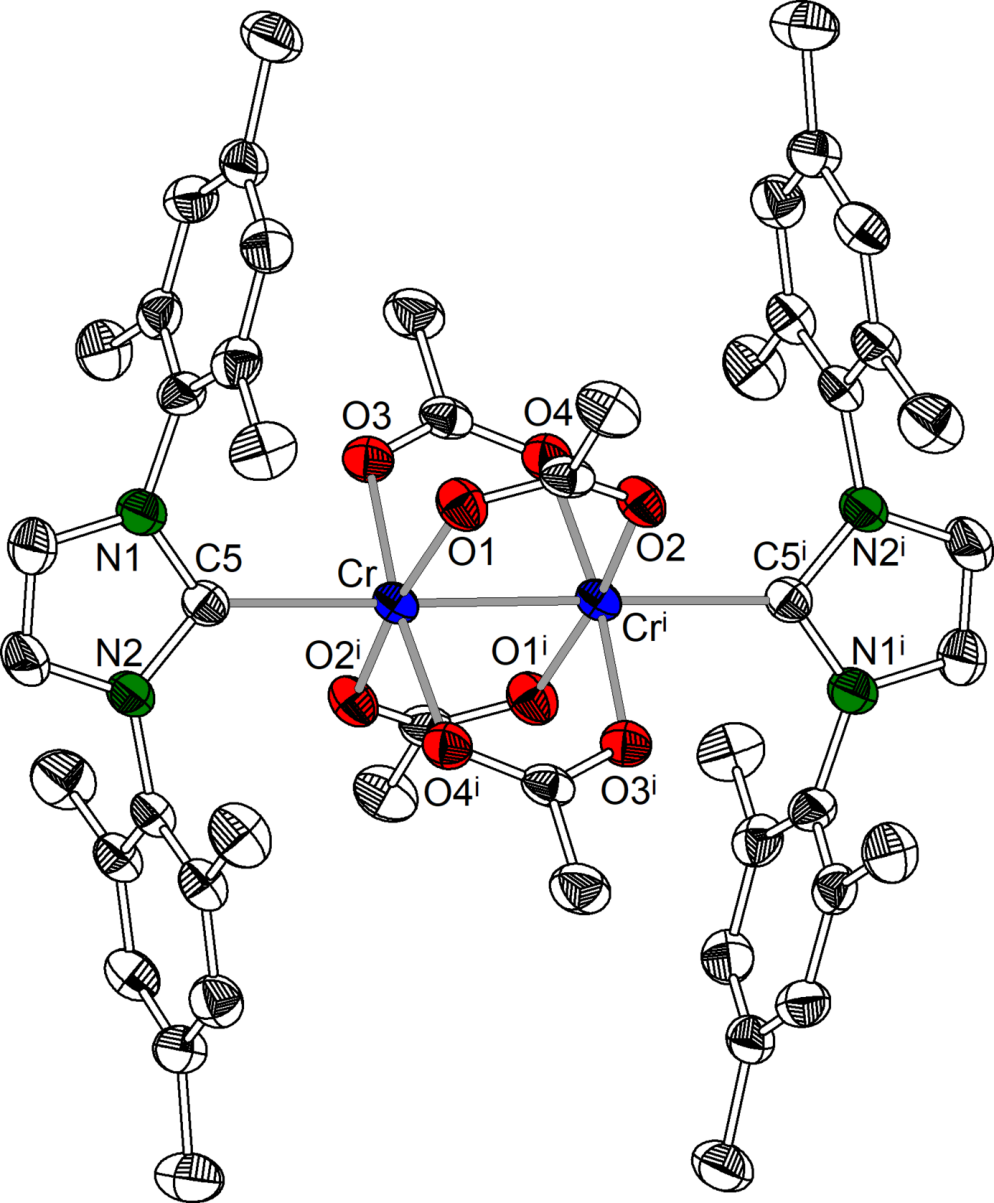
Mol­ecular structure of **2** in the crystal. Displacement ellipsoids are at the 50% probability level. H atoms are omitted for clarity. [Symmetry code: (i) −*x* + 1; −*y* + 1; −*z* + 1.

**Table 1:** Selected geometric parameters (Å, °) for **1**[Chem scheme1]

Cr—Cr^i^	2.5308 (6)	Cr—O3	2.0202 (13)
Cr—O1	2.0178 (14)	Cr—O4^i^	2.0248 (13)
Cr—O2^i^	2.0118 (14)	Cr—C5	2.3812 (16)
			
O1—Cr—O3	89.35 (6)	O2^i^—Cr—O4^i^	89.33 (6)
O1—Cr—O4^i^	90.32 (6)	O2^i^—Cr—C5	94.10 (6)
O1—Cr—C5	94.62 (6)	O3—Cr—O4^i^	171.54 (5)
O2^i^—Cr—O1	171.29 (5)	O3—Cr—C5	95.67 (5)
O2^i^—Cr—O3	89.72 (6)	O4^i^—Cr—C5	92.78 (5)

Symmetry code: (i) 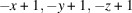.

**Table 2:** Selected geometric parameters (Å, °) for **2**[Chem scheme1]

Cr—Cr^i^	2.5284 (9)	Cr—O3	2.0269 (18)
Cr—O1	2.0274 (18)	Cr—O4^i^	2.0270 (18)
Cr—O2^i^	2.0238 (17)	Cr—C5	2.365 (3)
			
O1—Cr—C5	93.88 (8)	O3—Cr—O1	90.66 (8)
O2^i^—Cr—O1	171.65 (8)	O3—Cr—C5	93.90 (8)
O2^i^—Cr—O3	88.82 (7)	O4^i^—Cr—O1	88.28 (7)
O2^i^—Cr—O4^i^	91.03 (7)	O4^i^—Cr—O3	171.63 (8)
O2^i^—Cr—C5	94.46 (8)	O4^i^—Cr—C5	94.45 (8)

Symmetry code: (i) 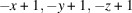.

**Table 3:** Hydrogen-bond geometry (Å, °) for **1**[Chem scheme1]

*D*—H⋯*A*	*D*—H	H⋯*A*	*D*⋯*A*	*D*—H⋯*A*
C6—H6⋯O5	0.95	2.47	3.411 (6)	171

**Table 4:** Hydrogen-bond geometry (Å, °) for **2**[Chem scheme1] *Cg* is the centroid of the C17–C22 ring.

*D*—H⋯*A*	*D*—H	H⋯*A*	*D*⋯*A*	*D*—H⋯*A*
C2—H2*B*⋯O2^ii^	0.98	2.61	3.527 (4)	155
C15—H15*A*⋯*Cg*^iii^	0.98	2.62	3.340 (3)	125

Symmetry codes: (ii) 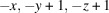; (iii) 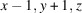.

**Table 5:** Experimental details

	**1**	**2**
Crystal data		
Chemical formula	[Cr_2_(C_2_H_3_O_2_)_4_(C_27_H_36_N_4_)_2_]·2C_4_H_8_O	{Cr_2_(C_2_H_3_O_2_)_4_(C_21_H_24_N_2_)_2_]
*M*_r_	1261.53	949.02
Crystal system, space group	Triclinic, *P* 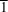	Triclinic, *P* 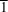
Temperature (K)	170	170
*a*, *b*, *c* (Å)	10.6402 (7), 11.7730 (8), 15.1884 (9)	8.3679 (5), 11.6127 (8), 13.8355 (9)
α, β, γ (°)	82.353 (5), 86.526 (5), 69.248 (5)	68.949 (5), 83.891 (5), 71.508 (5)
*V* (Å^3^)	1763.2 (2)	1189.90 (14)
*Z*	1	1
Radiation type	Mo *K*α	Mo *K*α
μ (mm^−1^)	0.37	0.51
Crystal size (mm)	0.61 × 0.35 × 0.15	0.40 × 0.24 × 0.07

Data collection		
Diffractometer	Stoe IPDS 2T	Stoe IPDS 2T
Absorption correction	Integration (*X-RED32*; Stoe & Cie, 2015 ▸)	Numerical (*X-RED32*; Stoe & Cie, 2015 ▸)
*T*_min_, *T*_max_	0.812, 0.947	0.844, 0.963
No. of measured, independent and observed [*I* > 2σ(*I*)] reflections	18724, 9444, 5824	8474, 4174, 2894
*R*_int_	0.070	0.071
(sin θ/λ)_max_ (Å^−1^)	0.687	0.595

Refinement		
*R*[*F*^2^ > 2σ(*F*^2^)], *wR*(*F*^2^), *S*	0.048, 0.133, 0.94	0.042, 0.112, 0.91
No. of reflections	9444	4174
No. of parameters	534	297
No. of restraints	545	0
H-atom treatment	H-atom parameters constrained	H-atom parameters constrained
Δρ_max_, Δρ_min_ (e Å^−3^)	0.47, −0.57	0.50, −0.51

Computer programs: *X-AREA* (Stoe & Cie, 2015 ▸), *SHELXT* (Sheldrick, 2015*a* ▸), *SHELXL* (Sheldrick, 2015*b* ▸), *OLEX2* (Dolomanov *et al.*, 2009 ▸) and *DIAMOND* (Brandenburg, 2019 ▸).

## supplementary crystallographic information

### Tetrakis(µ-acetato-κ2O:O')bis{[1,3-bis(2,6-diisopropylphenyl)imidazol-2-ylidene-κC2]chromium(II)} tetrahydrofuran disolvate (1) Crystal data

**Table:**

[Cr_2_(C_2_H_3_O_2_)_4_(C_27_H_36_N_4_)_2_]·2C_4_H_8_O	*Z* = 1
*M**_r_* = 1261.53	*F*(000) = 676
Triclinic, *P*1	*D*_x_ = 1.188 Mg m^−^^3^
*a* = 10.6402 (7) Å	Mo *K*α radiation, λ = 0.71073 Å
*b* = 11.7730 (8) Å	Cell parameters from 10431 reflections
*c* = 15.1884 (9) Å	θ = 1.9–29.5°
α = 82.353 (5)°	µ = 0.37 mm^−^^1^
β = 86.526 (5)°	*T* = 170 K
γ = 69.248 (5)°	Plate, clear violet
*V* = 1763.2 (2) Å^3^	0.61 × 0.35 × 0.15 mm

### Tetrakis(µ-acetato-κ2O:O')bis{[1,3-bis(2,6-diisopropylphenyl)imidazol-2-ylidene-κC2]chromium(II)} tetrahydrofuran disolvate (1) Data collection

**Table:**

Stoe IPDS 2T diffractometer	5824 reflections with *I* > 2σ(*I*)
Radiation source: sealed X-ray tube, 12 x 0.4 mm long-fine focus	*R*_int_ = 0.070
rotation method, ω scans	θ_max_ = 29.2°, θ_min_ = 2.2°
Absorption correction: integration (X-RED32; Stoe & Cie, 2015)	*h* = −14→12
*T*_min_ = 0.812, *T*_max_ = 0.947	*k* = −16→16
18724 measured reflections	*l* = −19→20
9444 independent reflections	

### Tetrakis(µ-acetato-κ2O:O')bis{[1,3-bis(2,6-diisopropylphenyl)imidazol-2-ylidene-κC2]chromium(II)} tetrahydrofuran disolvate (1) Refinement

**Table:**

Refinement on *F*^2^	Primary atom site location: iterative
Least-squares matrix: full	Hydrogen site location: inferred from neighbouring sites
*R*[*F*^2^ > 2σ(*F*^2^)] = 0.048	H-atom parameters constrained
*wR*(*F*^2^) = 0.133	*w* = 1/[σ^2^(*F*_o_^2^) + (0.071*P*)^2^] where *P* = (*F*_o_^2^ + 2*F*_c_^2^)/3
*S* = 0.94	(Δ/σ)_max_ = 0.001
9444 reflections	Δρ_max_ = 0.47 e Å^−^^3^
534 parameters	Δρ_min_ = −0.57 e Å^−^^3^
545 restraints	

### Tetrakis(µ-acetato-κ2O:O')bis{[1,3-bis(2,6-diisopropylphenyl)imidazol-2-ylidene-κC2]chromium(II)} tetrahydrofuran disolvate (1) Special details

**Table:**

Geometry. All esds (except the esd in the dihedral angle between two l.s. planes) are estimated using the full covariance matrix. The cell esds are taken into account individually in the estimation of esds in distances, angles and torsion angles; correlations between esds in cell parameters are only used when they are defined by crystal symmetry. An approximate (isotropic) treatment of cell esds is used for estimating esds involving l.s. planes.

### Tetrakis(µ-acetato-κ2O:O')bis{[1,3-bis(2,6-diisopropylphenyl)imidazol-2-ylidene-κC2]chromium(II)} tetrahydrofuran disolvate (1) Fractional atomic coordinates and isotropic or equivalent isotropic displacement parameters (Å^2^)

**Table:**

	*x*	*y*	*z*	*U*_iso_*/*U*_eq_	Occ. (<1)
Cr	0.51563 (3)	0.44550 (3)	0.57837 (2)	0.03006 (9)	
O1	0.39582 (14)	0.60752 (13)	0.61610 (8)	0.0403 (3)	
O2	0.36797 (14)	0.70259 (12)	0.47808 (8)	0.0403 (3)	
O3	0.67559 (14)	0.49924 (13)	0.58164 (8)	0.0406 (3)	
O4	0.64785 (14)	0.59629 (13)	0.44363 (8)	0.0399 (3)	
N1	0.60181 (15)	0.21883 (14)	0.75056 (9)	0.0325 (3)	
N2	0.48688 (15)	0.38398 (15)	0.80273 (9)	0.0329 (3)	
C1	0.70675 (19)	0.56408 (18)	0.51710 (12)	0.0354 (4)	
C2	0.8213 (2)	0.6063 (2)	0.52997 (15)	0.0506 (5)	
H2A	0.791711	0.671987	0.568287	0.076*	
H2B	0.850789	0.636891	0.472198	0.076*	
H2C	0.896176	0.537674	0.557824	0.076*	
C3	0.34728 (19)	0.70022 (18)	0.56080 (12)	0.0358 (4)	
C4	0.2567 (3)	0.8160 (2)	0.59504 (15)	0.0542 (6)	
H4A	0.294515	0.827236	0.649105	0.081*	
H4B	0.167515	0.810767	0.608564	0.081*	
H4C	0.248798	0.885719	0.549794	0.081*	
C5	0.54178 (17)	0.34020 (17)	0.72500 (10)	0.0278 (4)	
C6	0.5840 (2)	0.1883 (2)	0.84051 (13)	0.0505 (6)	
H6	0.616773	0.108775	0.872523	0.061*	
C7	0.5127 (2)	0.2907 (2)	0.87331 (13)	0.0499 (6)	
H7	0.484593	0.299120	0.933398	0.060*	
C8	0.67707 (19)	0.12560 (17)	0.69619 (12)	0.0344 (4)	
C9	0.8118 (2)	0.1107 (2)	0.67531 (13)	0.0414 (5)	
C17	0.8724 (12)	0.2037 (11)	0.6948 (7)	0.053 (2)	0.62 (4)
H17	0.796845	0.283453	0.697251	0.063*	0.62 (4)
C18	0.9722 (17)	0.2254 (17)	0.6252 (10)	0.087 (4)	0.62 (4)
H18A	0.998011	0.293219	0.638655	0.130*	0.62 (4)
H18B	0.930993	0.245723	0.566541	0.130*	0.62 (4)
H18C	1.052242	0.151178	0.625449	0.130*	0.62 (4)
C19	0.9351 (19)	0.1647 (16)	0.7874 (8)	0.086 (3)	0.62 (4)
H19A	1.007677	0.085133	0.787992	0.130*	0.62 (4)
H19B	0.866184	0.158430	0.831626	0.130*	0.62 (4)
H19C	0.971431	0.225750	0.801602	0.130*	0.62 (4)
C17A	0.8780 (19)	0.1943 (18)	0.7040 (11)	0.052 (4)	0.38 (4)
H17A	0.807541	0.266510	0.726863	0.063*	0.38 (4)
C18A	0.9500 (19)	0.2389 (19)	0.6223 (11)	0.049 (3)	0.38 (4)
H18D	1.015024	0.168102	0.597399	0.074*	0.38 (4)
H18E	0.996975	0.289880	0.640528	0.074*	0.38 (4)
H18F	0.883495	0.286990	0.577257	0.074*	0.38 (4)
C19A	0.984 (3)	0.130 (3)	0.7761 (13)	0.090 (5)	0.38 (4)
H19D	0.942427	0.097741	0.828075	0.135*	0.38 (4)
H19E	1.022201	0.187942	0.793422	0.135*	0.38 (4)
H19F	1.056381	0.061689	0.752591	0.135*	0.38 (4)
C10	0.8862 (2)	0.0104 (2)	0.63229 (16)	0.0546 (6)	
H10	0.978136	−0.003250	0.618022	0.065*	
C11	0.8284 (3)	−0.0701 (2)	0.60989 (16)	0.0592 (6)	
H11	0.881398	−0.139428	0.581869	0.071*	
C12	0.6939 (3)	−0.0501 (2)	0.62810 (16)	0.0542 (6)	
H12	0.654747	−0.104574	0.610834	0.065*	
C13	0.6155 (2)	0.04846 (19)	0.67118 (13)	0.0418 (5)	
C14	0.4661 (2)	0.0731 (2)	0.68671 (17)	0.0553 (6)	
H14	0.427192	0.150232	0.715257	0.066*	
C15	0.3947 (3)	0.0925 (3)	0.59920 (19)	0.0694 (8)	
H15A	0.427315	0.016458	0.571509	0.104*	
H15B	0.413040	0.157596	0.559385	0.104*	
H15C	0.297612	0.115795	0.610327	0.104*	
C16	0.4421 (3)	−0.0301 (3)	0.74994 (18)	0.0730 (8)	
H16A	0.478971	−0.106792	0.723250	0.110*	
H16B	0.345402	−0.010425	0.760669	0.110*	
H16C	0.486728	−0.039199	0.806386	0.110*	
C20	0.41683 (19)	0.50940 (18)	0.81624 (11)	0.0337 (4)	
C21	0.4917 (2)	0.5764 (2)	0.83985 (12)	0.0396 (4)	
C29	0.6447 (4)	0.5264 (7)	0.8436 (5)	0.0494 (14)	0.81 (3)
H29	0.676101	0.441254	0.826911	0.059*	0.81 (3)
C30	0.6974 (6)	0.5195 (11)	0.9357 (3)	0.0653 (17)	0.81 (3)
H30A	0.657407	0.471795	0.978742	0.098*	0.81 (3)
H30B	0.673263	0.602289	0.952331	0.098*	0.81 (3)
H30C	0.795382	0.479886	0.935514	0.098*	0.81 (3)
C31	0.7034 (9)	0.6025 (12)	0.7751 (6)	0.077 (3)	0.81 (3)
H31A	0.801758	0.568940	0.777457	0.115*	0.81 (3)
H31B	0.671341	0.687438	0.788361	0.115*	0.81 (3)
H31C	0.674649	0.599894	0.715505	0.115*	0.81 (3)
C29A	0.6444 (16)	0.521 (3)	0.8323 (18)	0.050 (5)	0.19 (3)
H29A	0.668222	0.454919	0.792796	0.060*	0.19 (3)
C30A	0.702 (2)	0.465 (4)	0.9241 (18)	0.065 (6)	0.19 (3)
H30D	0.681521	0.529102	0.963094	0.098*	0.19 (3)
H30E	0.799164	0.424276	0.919044	0.098*	0.19 (3)
H30F	0.660800	0.404589	0.949035	0.098*	0.19 (3)
C31A	0.702 (3)	0.618 (4)	0.791 (3)	0.059 (6)	0.19 (3)
H31D	0.798735	0.589120	0.802004	0.089*	0.19 (3)
H31E	0.657419	0.694141	0.817389	0.089*	0.19 (3)
H31F	0.686969	0.633217	0.726704	0.089*	0.19 (3)
C22	0.4213 (2)	0.6941 (2)	0.86010 (14)	0.0492 (5)	
H22	0.469330	0.742251	0.876560	0.059*	
C23	0.2827 (3)	0.7426 (2)	0.85676 (15)	0.0535 (6)	
H23	0.235827	0.822664	0.872055	0.064*	
C24	0.2127 (2)	0.6741 (2)	0.83113 (15)	0.0525 (6)	
H24	0.117536	0.708661	0.827874	0.063*	
C25	0.2774 (2)	0.5564 (2)	0.81002 (13)	0.0415 (5)	
C26	0.2014 (13)	0.4846 (12)	0.7750 (10)	0.047 (2)	0.60 (4)
H26	0.266183	0.414906	0.746052	0.056*	0.60 (4)
C27	0.134 (2)	0.4341 (18)	0.8561 (11)	0.075 (4)	0.60 (4)
H27A	0.078191	0.501764	0.888165	0.112*	0.60 (4)
H27B	0.204055	0.376262	0.895663	0.112*	0.60 (4)
H27C	0.078602	0.392077	0.836074	0.112*	0.60 (4)
C28	0.0943 (17)	0.5648 (19)	0.7081 (10)	0.058 (3)	0.60 (4)
H28A	0.046268	0.515840	0.688085	0.087*	0.60 (4)
H28B	0.137570	0.596961	0.656950	0.087*	0.60 (4)
H28C	0.030568	0.633000	0.736433	0.087*	0.60 (4)
C26A	0.1949 (17)	0.4790 (18)	0.7944 (15)	0.052 (3)	0.40 (4)
H26A	0.260668	0.397623	0.781853	0.063*	0.40 (4)
C27A	0.106 (2)	0.453 (2)	0.8714 (16)	0.068 (4)	0.40 (4)
H27D	0.161822	0.409929	0.922812	0.103*	0.40 (4)
H27E	0.057904	0.402910	0.853662	0.103*	0.40 (4)
H27F	0.040754	0.530928	0.887135	0.103*	0.40 (4)
C28A	0.110 (3)	0.533 (3)	0.7104 (15)	0.059 (4)	0.40 (4)
H28D	0.067387	0.476743	0.696607	0.088*	0.40 (4)
H28E	0.168554	0.546182	0.660451	0.088*	0.40 (4)
H28F	0.041091	0.611738	0.720449	0.088*	0.40 (4)
O5	0.6804 (4)	−0.1070 (5)	0.9384 (3)	0.0790 (11)	0.795 (12)
C32	0.6950 (7)	−0.0781 (10)	1.0249 (6)	0.0881 (18)	0.795 (12)
H32A	0.708247	−0.149901	1.070183	0.106*	0.795 (12)
H32B	0.615284	−0.009512	1.041729	0.106*	0.795 (12)
C33	0.8176 (5)	−0.0426 (5)	1.0150 (3)	0.0768 (14)	0.795 (12)
H33A	0.856158	−0.045779	1.073360	0.092*	0.795 (12)
H33B	0.798160	0.040072	0.982139	0.092*	0.795 (12)
C34	0.9097 (5)	−0.1411 (6)	0.9618 (4)	0.0849 (18)	0.795 (12)
H34A	0.977508	−0.113312	0.927105	0.102*	0.795 (12)
H34B	0.956073	−0.217464	1.000710	0.102*	0.795 (12)
C35	0.8117 (5)	−0.1581 (5)	0.9022 (3)	0.0747 (14)	0.795 (12)
H35A	0.817227	−0.116739	0.841648	0.090*	0.795 (12)
H35B	0.833126	−0.246289	0.898199	0.090*	0.795 (12)
O5A	0.6961 (19)	−0.152 (2)	0.9615 (14)	0.091 (4)	0.205 (12)
C32A	0.686 (2)	−0.067 (4)	1.022 (3)	0.088 (5)	0.205 (12)
H32C	0.638742	−0.083928	1.077817	0.106*	0.205 (12)
H32D	0.638579	0.018375	0.995494	0.106*	0.205 (12)
C33A	0.832 (2)	−0.091 (2)	1.0371 (13)	0.092 (4)	0.205 (12)
H33C	0.872091	−0.166299	1.078330	0.110*	0.205 (12)
H33D	0.844468	−0.021169	1.060362	0.110*	0.205 (12)
C34A	0.889 (2)	−0.105 (2)	0.9437 (15)	0.087 (4)	0.205 (12)
H34C	0.857479	−0.027788	0.903504	0.104*	0.205 (12)
H34D	0.988378	−0.139980	0.942702	0.104*	0.205 (12)
C35A	0.827 (2)	−0.195 (2)	0.9227 (17)	0.086 (4)	0.205 (12)
H35C	0.822488	−0.195092	0.857796	0.103*	0.205 (12)
H35D	0.878724	−0.279412	0.949626	0.103*	0.205 (12)

### Tetrakis(µ-acetato-κ2O:O')bis{[1,3-bis(2,6-diisopropylphenyl)imidazol-2-ylidene-κC2]chromium(II)} tetrahydrofuran disolvate (1) Atomic displacement parameters (Å^2^)

**Table:**

	*U*^11^	*U*^22^	*U*^33^	*U*^12^	*U*^13^	*U*^23^
Cr	0.02749 (16)	0.03361 (17)	0.02747 (15)	−0.00935 (12)	−0.00114 (10)	−0.00153 (11)
O1	0.0433 (8)	0.0381 (8)	0.0316 (7)	−0.0049 (6)	0.0010 (5)	−0.0038 (5)
O2	0.0421 (8)	0.0373 (8)	0.0327 (7)	−0.0040 (6)	0.0002 (5)	−0.0019 (5)
O3	0.0367 (8)	0.0499 (9)	0.0379 (7)	−0.0203 (7)	−0.0063 (5)	0.0026 (6)
O4	0.0379 (8)	0.0501 (9)	0.0350 (7)	−0.0217 (7)	−0.0040 (5)	0.0029 (6)
N1	0.0304 (8)	0.0357 (9)	0.0281 (7)	−0.0085 (7)	0.0002 (6)	−0.0004 (6)
N2	0.0320 (8)	0.0387 (9)	0.0260 (7)	−0.0101 (7)	−0.0016 (6)	−0.0026 (6)
C1	0.0285 (10)	0.0376 (11)	0.0395 (10)	−0.0107 (8)	0.0001 (7)	−0.0052 (8)
C2	0.0390 (12)	0.0618 (15)	0.0565 (12)	−0.0261 (12)	−0.0054 (9)	−0.0007 (11)
C3	0.0308 (10)	0.0381 (11)	0.0380 (10)	−0.0111 (9)	0.0014 (7)	−0.0069 (8)
C4	0.0583 (15)	0.0422 (13)	0.0497 (12)	−0.0016 (11)	0.0042 (10)	−0.0104 (10)
C5	0.0236 (9)	0.0323 (9)	0.0270 (8)	−0.0088 (7)	−0.0006 (6)	−0.0050 (7)
C6	0.0554 (14)	0.0460 (13)	0.0348 (10)	−0.0033 (11)	0.0009 (9)	0.0069 (9)
C7	0.0609 (15)	0.0517 (14)	0.0251 (9)	−0.0086 (12)	0.0021 (9)	0.0045 (8)
C8	0.0320 (10)	0.0308 (10)	0.0349 (9)	−0.0057 (8)	−0.0016 (7)	0.0006 (7)
C9	0.0344 (11)	0.0406 (11)	0.0422 (10)	−0.0067 (9)	−0.0029 (8)	0.0015 (8)
C17	0.039 (4)	0.055 (4)	0.064 (4)	−0.014 (3)	−0.008 (3)	−0.011 (3)
C18	0.051 (6)	0.101 (9)	0.114 (7)	−0.038 (6)	0.019 (5)	−0.009 (6)
C19	0.089 (8)	0.105 (7)	0.087 (5)	−0.061 (7)	−0.032 (5)	0.002 (4)
C17A	0.035 (6)	0.065 (7)	0.058 (6)	−0.024 (5)	0.010 (4)	0.005 (5)
C18A	0.026 (4)	0.054 (6)	0.065 (6)	−0.017 (4)	−0.002 (3)	0.010 (5)
C19A	0.086 (10)	0.138 (13)	0.060 (6)	−0.063 (9)	−0.025 (6)	0.017 (6)
C10	0.0359 (12)	0.0528 (14)	0.0653 (14)	−0.0040 (11)	0.0051 (10)	−0.0086 (11)
C11	0.0550 (15)	0.0468 (14)	0.0626 (14)	0.0005 (12)	0.0055 (11)	−0.0154 (11)
C12	0.0535 (14)	0.0424 (13)	0.0657 (14)	−0.0129 (11)	−0.0036 (11)	−0.0126 (11)
C13	0.0384 (11)	0.0363 (11)	0.0471 (11)	−0.0100 (9)	−0.0015 (8)	−0.0001 (9)
C14	0.0412 (13)	0.0545 (15)	0.0772 (15)	−0.0217 (11)	0.0028 (11)	−0.0194 (12)
C15	0.0515 (15)	0.0661 (18)	0.092 (2)	−0.0258 (14)	−0.0218 (14)	0.0107 (15)
C16	0.0687 (19)	0.097 (2)	0.0678 (16)	−0.0495 (18)	0.0039 (13)	−0.0028 (15)
C20	0.0357 (10)	0.0404 (11)	0.0230 (8)	−0.0111 (9)	0.0029 (7)	−0.0047 (7)
C21	0.0404 (11)	0.0484 (12)	0.0333 (9)	−0.0190 (10)	0.0068 (7)	−0.0098 (8)
C29	0.037 (2)	0.066 (3)	0.052 (3)	−0.022 (2)	0.0076 (18)	−0.023 (2)
C30	0.049 (2)	0.100 (5)	0.049 (2)	−0.031 (3)	−0.0036 (15)	−0.003 (2)
C31	0.061 (3)	0.144 (6)	0.048 (3)	−0.063 (4)	0.014 (2)	−0.017 (3)
C29A	0.042 (9)	0.077 (10)	0.042 (7)	−0.025 (7)	−0.014 (6)	−0.023 (7)
C30A	0.045 (8)	0.091 (15)	0.061 (9)	−0.026 (10)	−0.005 (6)	−0.003 (10)
C31A	0.043 (10)	0.088 (11)	0.050 (12)	−0.026 (9)	0.002 (8)	−0.014 (9)
C22	0.0551 (14)	0.0531 (14)	0.0475 (11)	−0.0267 (12)	0.0113 (10)	−0.0175 (10)
C23	0.0563 (15)	0.0455 (13)	0.0552 (13)	−0.0111 (12)	0.0115 (11)	−0.0186 (10)
C24	0.0381 (12)	0.0549 (14)	0.0566 (13)	−0.0048 (11)	0.0026 (10)	−0.0135 (11)
C25	0.0340 (11)	0.0506 (13)	0.0371 (10)	−0.0092 (10)	0.0002 (8)	−0.0112 (9)
C26	0.039 (3)	0.061 (4)	0.045 (4)	−0.019 (3)	−0.007 (3)	−0.012 (3)
C27	0.079 (8)	0.094 (7)	0.070 (6)	−0.057 (7)	−0.025 (5)	0.009 (5)
C28	0.037 (4)	0.082 (8)	0.053 (4)	−0.018 (4)	−0.011 (3)	−0.003 (4)
C26A	0.027 (4)	0.065 (6)	0.059 (7)	−0.002 (4)	−0.001 (4)	−0.026 (5)
C27A	0.057 (6)	0.081 (7)	0.069 (7)	−0.032 (6)	0.003 (5)	0.003 (6)
C28A	0.039 (7)	0.082 (11)	0.057 (6)	−0.020 (7)	0.004 (4)	−0.018 (6)
O5	0.0649 (19)	0.076 (3)	0.094 (2)	−0.0215 (19)	−0.0176 (16)	−0.0061 (19)
C32	0.080 (3)	0.101 (4)	0.062 (3)	−0.011 (3)	0.015 (2)	0.002 (3)
C33	0.080 (3)	0.076 (3)	0.067 (3)	−0.012 (2)	−0.026 (2)	−0.013 (2)
C34	0.057 (3)	0.087 (4)	0.107 (4)	−0.018 (3)	0.002 (2)	−0.021 (3)
C35	0.082 (3)	0.063 (3)	0.072 (3)	−0.013 (2)	−0.004 (2)	−0.017 (2)
O5A	0.070 (6)	0.095 (8)	0.103 (8)	−0.025 (6)	−0.006 (5)	−0.006 (6)
C32A	0.076 (7)	0.092 (8)	0.078 (8)	−0.008 (7)	−0.009 (7)	−0.002 (7)
C33A	0.083 (7)	0.087 (8)	0.091 (7)	−0.006 (7)	−0.024 (6)	−0.021 (7)
C34A	0.071 (7)	0.073 (8)	0.110 (7)	−0.017 (6)	−0.001 (7)	−0.011 (7)
C35A	0.081 (7)	0.073 (8)	0.096 (8)	−0.017 (6)	0.000 (6)	−0.017 (7)

### Tetrakis(µ-acetato-κ2O:O')bis{[1,3-bis(2,6-diisopropylphenyl)imidazol-2-ylidene-κC2]chromium(II)} tetrahydrofuran disolvate (1) Geometric parameters (Å, º)

**Table:**

Cr—Cr^i^	2.5308 (6)	C29—H29	1.0000
Cr—O1	2.0178 (14)	C29—C30	1.520 (6)
Cr—O2^i^	2.0118 (14)	C29—C31	1.532 (6)
Cr—O3	2.0202 (13)	C30—H30A	0.9800
Cr—O4^i^	2.0248 (13)	C30—H30B	0.9800
Cr—C5	2.3812 (16)	C30—H30C	0.9800
O1—C3	1.254 (2)	C31—H31A	0.9800
O2—C3	1.261 (2)	C31—H31B	0.9800
O3—C1	1.263 (2)	C31—H31C	0.9800
O4—C1	1.259 (2)	C29A—H29A	1.0000
N1—C5	1.355 (2)	C29A—C30A	1.525 (15)
N1—C6	1.385 (2)	C29A—C31A	1.526 (15)
N1—C8	1.437 (2)	C30A—H30D	0.9800
N2—C5	1.366 (2)	C30A—H30E	0.9800
N2—C7	1.393 (2)	C30A—H30F	0.9800
N2—C20	1.435 (2)	C31A—H31D	0.9800
C1—C2	1.502 (3)	C31A—H31E	0.9800
C2—H2A	0.9800	C31A—H31F	0.9800
C2—H2B	0.9800	C22—H22	0.9500
C2—H2C	0.9800	C22—C23	1.382 (3)
C3—C4	1.502 (3)	C23—H23	0.9500
C4—H4A	0.9800	C23—C24	1.379 (3)
C4—H4B	0.9800	C24—H24	0.9500
C4—H4C	0.9800	C24—C25	1.383 (3)
C6—H6	0.9500	C25—C26	1.519 (8)
C6—C7	1.320 (3)	C25—C26A	1.519 (12)
C7—H7	0.9500	C26—H26	1.0000
C8—C9	1.403 (3)	C26—C27	1.542 (8)
C8—C13	1.394 (3)	C26—C28	1.531 (9)
C9—C17	1.521 (8)	C27—H27A	0.9800
C9—C17A	1.516 (12)	C27—H27B	0.9800
C9—C10	1.389 (3)	C27—H27C	0.9800
C17—H17	1.0000	C28—H28A	0.9800
C17—C18	1.514 (9)	C28—H28B	0.9800
C17—C19	1.535 (8)	C28—H28C	0.9800
C18—H18A	0.9800	C26A—H26A	1.0000
C18—H18B	0.9800	C26A—C27A	1.527 (11)
C18—H18C	0.9800	C26A—C28A	1.536 (11)
C19—H19A	0.9800	C27A—H27D	0.9800
C19—H19B	0.9800	C27A—H27E	0.9800
C19—H19C	0.9800	C27A—H27F	0.9800
C17A—H17A	1.0000	C28A—H28D	0.9800
C17A—C18A	1.549 (11)	C28A—H28E	0.9800
C17A—C19A	1.543 (12)	C28A—H28F	0.9800
C18A—H18D	0.9800	O5—C32	1.430 (7)
C18A—H18E	0.9800	O5—C35	1.420 (5)
C18A—H18F	0.9800	C32—H32A	0.9900
C19A—H19D	0.9800	C32—H32B	0.9900
C19A—H19E	0.9800	C32—C33	1.499 (6)
C19A—H19F	0.9800	C33—H33A	0.9900
C10—H10	0.9500	C33—H33B	0.9900
C10—C11	1.384 (4)	C33—C34	1.518 (5)
C11—H11	0.9500	C34—H34A	0.9900
C11—C12	1.382 (4)	C34—H34B	0.9900
C12—H12	0.9500	C34—C35	1.503 (5)
C12—C13	1.386 (3)	C35—H35A	0.9900
C13—C14	1.521 (3)	C35—H35B	0.9900
C14—H14	1.0000	O5A—C32A	1.421 (15)
C14—C15	1.522 (3)	O5A—C35A	1.423 (14)
C14—C16	1.531 (4)	C32A—H32C	0.9900
C15—H15A	0.9800	C32A—H32D	0.9900
C15—H15B	0.9800	C32A—C33A	1.504 (8)
C15—H15C	0.9800	C33A—H33C	0.9900
C16—H16A	0.9800	C33A—H33D	0.9900
C16—H16B	0.9800	C33A—C34A	1.517 (8)
C16—H16C	0.9800	C34A—H34C	0.9900
C20—C21	1.394 (3)	C34A—H34D	0.9900
C20—C25	1.392 (3)	C34A—C35A	1.505 (8)
C21—C29	1.524 (5)	C35A—H35C	0.9900
C21—C29A	1.525 (17)	C35A—H35D	0.9900
C21—C22	1.387 (3)		
			
O1—Cr—Cr^i^	85.85 (4)	C21—C29—C31	110.1 (5)
O1—Cr—O3	89.35 (6)	C30—C29—C21	113.0 (4)
O1—Cr—O4^i^	90.32 (6)	C30—C29—H29	107.6
O1—Cr—C5	94.62 (6)	C30—C29—C31	110.7 (5)
O2^i^—Cr—Cr^i^	85.44 (4)	C31—C29—H29	107.6
O2^i^—Cr—O1	171.29 (5)	C29—C30—H30A	109.5
O2^i^—Cr—O3	89.72 (6)	C29—C30—H30B	109.5
O2^i^—Cr—O4^i^	89.33 (6)	C29—C30—H30C	109.5
O2^i^—Cr—C5	94.10 (6)	H30A—C30—H30B	109.5
O3—Cr—Cr^i^	85.65 (4)	H30A—C30—H30C	109.5
O3—Cr—O4^i^	171.54 (5)	H30B—C30—H30C	109.5
O3—Cr—C5	95.67 (5)	C29—C31—H31A	109.5
O4^i^—Cr—Cr^i^	85.90 (4)	C29—C31—H31B	109.5
O4^i^—Cr—C5	92.78 (5)	C29—C31—H31C	109.5
C5—Cr—Cr^i^	178.60 (5)	H31A—C31—H31B	109.5
C3—O1—Cr	121.81 (12)	H31A—C31—H31C	109.5
C3—O2—Cr^i^	122.43 (13)	H31B—C31—H31C	109.5
C1—O3—Cr	122.10 (12)	C21—C29A—H29A	108.5
C1—O4—Cr^i^	121.67 (12)	C21—C29A—C30A	109.1 (16)
C5—N1—C6	112.15 (16)	C21—C29A—C31A	110 (2)
C5—N1—C8	127.76 (14)	C30A—C29A—H29A	108.5
C6—N1—C8	120.09 (16)	C30A—C29A—C31A	112 (2)
C5—N2—C7	111.56 (16)	C31A—C29A—H29A	108.5
C5—N2—C20	127.10 (14)	C29A—C30A—H30D	109.5
C7—N2—C20	121.26 (15)	C29A—C30A—H30E	109.5
O3—C1—C2	117.20 (17)	C29A—C30A—H30F	109.5
O4—C1—O3	124.59 (17)	H30D—C30A—H30E	109.5
O4—C1—C2	118.21 (17)	H30D—C30A—H30F	109.5
C1—C2—H2A	109.5	H30E—C30A—H30F	109.5
C1—C2—H2B	109.5	C29A—C31A—H31D	109.5
C1—C2—H2C	109.5	C29A—C31A—H31E	109.5
H2A—C2—H2B	109.5	C29A—C31A—H31F	109.5
H2A—C2—H2C	109.5	H31D—C31A—H31E	109.5
H2B—C2—H2C	109.5	H31D—C31A—H31F	109.5
O1—C3—O2	124.46 (18)	H31E—C31A—H31F	109.5
O1—C3—C4	117.95 (17)	C21—C22—H22	119.4
O2—C3—C4	117.58 (18)	C23—C22—C21	121.2 (2)
C3—C4—H4A	109.5	C23—C22—H22	119.4
C3—C4—H4B	109.5	C22—C23—H23	120.2
C3—C4—H4C	109.5	C24—C23—C22	119.6 (2)
H4A—C4—H4B	109.5	C24—C23—H23	120.2
H4A—C4—H4C	109.5	C23—C24—H24	119.1
H4B—C4—H4C	109.5	C23—C24—C25	121.8 (2)
N1—C5—Cr	128.25 (12)	C25—C24—H24	119.1
N1—C5—N2	102.64 (14)	C20—C25—C26	121.1 (6)
N2—C5—Cr	128.71 (13)	C20—C25—C26A	122.9 (8)
N1—C6—H6	126.5	C24—C25—C20	117.0 (2)
C7—C6—N1	106.96 (18)	C24—C25—C26	121.7 (6)
C7—C6—H6	126.5	C24—C25—C26A	119.5 (8)
N2—C7—H7	126.7	C25—C26—H26	109.4
C6—C7—N2	106.69 (17)	C25—C26—C27	106.7 (8)
C6—C7—H7	126.7	C25—C26—C28	111.9 (9)
C9—C8—N1	118.88 (18)	C27—C26—H26	109.4
C13—C8—N1	118.39 (17)	C28—C26—H26	109.4
C13—C8—C9	122.60 (19)	C28—C26—C27	110.0 (10)
C8—C9—C17	121.5 (5)	C26—C27—H27A	109.5
C8—C9—C17A	122.0 (8)	C26—C27—H27B	109.5
C10—C9—C8	117.3 (2)	C26—C27—H27C	109.5
C10—C9—C17	121.2 (5)	H27A—C27—H27B	109.5
C10—C9—C17A	120.6 (8)	H27A—C27—H27C	109.5
C9—C17—H17	107.3	H27B—C27—H27C	109.5
C9—C17—C19	108.8 (8)	C26—C28—H28A	109.5
C18—C17—C9	114.5 (9)	C26—C28—H28B	109.5
C18—C17—H17	107.3	C26—C28—H28C	109.5
C18—C17—C19	111.4 (9)	H28A—C28—H28B	109.5
C19—C17—H17	107.3	H28A—C28—H28C	109.5
C17—C18—H18A	109.5	H28B—C28—H28C	109.5
C17—C18—H18B	109.5	C25—C26A—H26A	106.4
C17—C18—H18C	109.5	C25—C26A—C27A	116.8 (14)
H18A—C18—H18B	109.5	C25—C26A—C28A	110.5 (13)
H18A—C18—H18C	109.5	C27A—C26A—H26A	106.4
H18B—C18—H18C	109.5	C27A—C26A—C28A	109.7 (14)
C17—C19—H19A	109.5	C28A—C26A—H26A	106.4
C17—C19—H19B	109.5	C26A—C27A—H27D	109.5
C17—C19—H19C	109.5	C26A—C27A—H27E	109.5
H19A—C19—H19B	109.5	C26A—C27A—H27F	109.5
H19A—C19—H19C	109.5	H27D—C27A—H27E	109.5
H19B—C19—H19C	109.5	H27D—C27A—H27F	109.5
C9—C17A—H17A	109.3	H27E—C27A—H27F	109.5
C9—C17A—C18A	108.6 (12)	C26A—C28A—H28D	109.5
C9—C17A—C19A	112.7 (13)	C26A—C28A—H28E	109.5
C18A—C17A—H17A	109.3	C26A—C28A—H28F	109.5
C19A—C17A—H17A	109.3	H28D—C28A—H28E	109.5
C19A—C17A—C18A	107.5 (12)	H28D—C28A—H28F	109.5
C17A—C18A—H18D	109.5	H28E—C28A—H28F	109.5
C17A—C18A—H18E	109.5	C35—O5—C32	107.2 (4)
C17A—C18A—H18F	109.5	O5—C32—H32A	111.1
H18D—C18A—H18E	109.5	O5—C32—H32B	111.1
H18D—C18A—H18F	109.5	O5—C32—C33	103.4 (5)
H18E—C18A—H18F	109.5	H32A—C32—H32B	109.1
C17A—C19A—H19D	109.5	C33—C32—H32A	111.1
C17A—C19A—H19E	109.5	C33—C32—H32B	111.1
C17A—C19A—H19F	109.5	C32—C33—H33A	111.6
H19D—C19A—H19E	109.5	C32—C33—H33B	111.6
H19D—C19A—H19F	109.5	C32—C33—C34	100.9 (5)
H19E—C19A—H19F	109.5	H33A—C33—H33B	109.4
C9—C10—H10	119.5	C34—C33—H33A	111.6
C11—C10—C9	121.0 (2)	C34—C33—H33B	111.6
C11—C10—H10	119.5	C33—C34—H34A	111.4
C10—C11—H11	119.9	C33—C34—H34B	111.4
C12—C11—C10	120.3 (2)	H34A—C34—H34B	109.3
C12—C11—H11	119.9	C35—C34—C33	101.6 (4)
C11—C12—H12	119.5	C35—C34—H34A	111.4
C11—C12—C13	120.9 (2)	C35—C34—H34B	111.4
C13—C12—H12	119.5	O5—C35—C34	108.3 (3)
C8—C13—C14	121.80 (19)	O5—C35—H35A	110.0
C12—C13—C8	117.8 (2)	O5—C35—H35B	110.0
C12—C13—C14	120.4 (2)	C34—C35—H35A	110.0
C13—C14—H14	107.9	C34—C35—H35B	110.0
C13—C14—C15	110.9 (2)	H35A—C35—H35B	108.4
C13—C14—C16	111.1 (2)	C32A—O5A—C35A	111.9 (14)
C15—C14—H14	107.9	O5A—C32A—H32C	111.6
C15—C14—C16	110.9 (2)	O5A—C32A—H32D	111.6
C16—C14—H14	107.9	O5A—C32A—C33A	100.7 (14)
C14—C15—H15A	109.5	H32C—C32A—H32D	109.4
C14—C15—H15B	109.5	C33A—C32A—H32C	111.6
C14—C15—H15C	109.5	C33A—C32A—H32D	111.6
H15A—C15—H15B	109.5	C32A—C33A—H33C	111.5
H15A—C15—H15C	109.5	C32A—C33A—H33D	111.5
H15B—C15—H15C	109.5	C32A—C33A—C34A	101.1 (15)
C14—C16—H16A	109.5	H33C—C33A—H33D	109.4
C14—C16—H16B	109.5	C34A—C33A—H33C	111.5
C14—C16—H16C	109.5	C34A—C33A—H33D	111.5
H16A—C16—H16B	109.5	C33A—C34A—H34C	112.3
H16A—C16—H16C	109.5	C33A—C34A—H34D	112.3
H16B—C16—H16C	109.5	H34C—C34A—H34D	109.9
C21—C20—N2	118.01 (17)	C35A—C34A—C33A	97.4 (13)
C25—C20—N2	118.83 (18)	C35A—C34A—H34C	112.3
C25—C20—C21	123.06 (19)	C35A—C34A—H34D	112.3
C20—C21—C29	123.0 (4)	O5A—C35A—C34A	103.3 (13)
C20—C21—C29A	118.0 (14)	O5A—C35A—H35C	111.1
C22—C21—C20	117.27 (19)	O5A—C35A—H35D	111.1
C22—C21—C29	119.7 (4)	C34A—C35A—H35C	111.1
C22—C21—C29A	124.4 (14)	C34A—C35A—H35D	111.1
C21—C29—H29	107.6	H35C—C35A—H35D	109.1
			
Cr—O1—C3—O2	−0.4 (3)	C10—C9—C17A—C19A	−65.1 (17)
Cr—O1—C3—C4	178.64 (15)	C10—C11—C12—C13	−1.8 (4)
Cr^i^—O2—C3—O1	0.1 (3)	C11—C12—C13—C8	−0.6 (3)
Cr^i^—O2—C3—C4	−179.01 (14)	C11—C12—C13—C14	176.9 (2)
Cr—O3—C1—O4	−3.9 (3)	C12—C13—C14—C15	−58.8 (3)
Cr—O3—C1—C2	175.42 (14)	C12—C13—C14—C16	65.2 (3)
Cr^i^—O4—C1—O3	3.5 (3)	C13—C8—C9—C17	173.5 (5)
Cr^i^—O4—C1—C2	−175.80 (14)	C13—C8—C9—C17A	−179.2 (8)
N1—C6—C7—N2	0.1 (3)	C13—C8—C9—C10	−3.6 (3)
N1—C8—C9—C17	−10.8 (6)	C20—N2—C5—Cr	−10.6 (3)
N1—C8—C9—C17A	−3.5 (8)	C20—N2—C5—N1	176.26 (16)
N1—C8—C9—C10	172.11 (18)	C20—N2—C7—C6	−176.71 (19)
N1—C8—C13—C12	−172.31 (18)	C20—C21—C29—C30	−118.9 (6)
N1—C8—C13—C14	10.2 (3)	C20—C21—C29—C31	116.7 (6)
N2—C20—C21—C29	5.7 (4)	C20—C21—C29A—C30A	−100 (3)
N2—C20—C21—C29A	11.7 (11)	C20—C21—C29A—C31A	137 (2)
N2—C20—C21—C22	−174.60 (16)	C20—C21—C22—C23	−0.1 (3)
N2—C20—C25—C24	174.45 (17)	C20—C25—C26—C27	105.0 (10)
N2—C20—C25—C26	−10.0 (7)	C20—C25—C26—C28	−134.6 (11)
N2—C20—C25—C26A	3.4 (10)	C20—C25—C26A—C27A	111.0 (15)
C5—N1—C6—C7	−0.4 (3)	C20—C25—C26A—C28A	−122.7 (14)
C5—N1—C8—C9	80.8 (2)	C21—C20—C25—C24	−1.9 (3)
C5—N1—C8—C13	−103.4 (2)	C21—C20—C25—C26	173.7 (7)
C5—N2—C7—C6	0.1 (2)	C21—C20—C25—C26A	−173.0 (10)
C5—N2—C20—C21	−90.0 (2)	C21—C22—C23—C24	−1.4 (3)
C5—N2—C20—C25	93.4 (2)	C29—C21—C22—C23	179.6 (3)
C6—N1—C5—Cr	−172.69 (14)	C29A—C21—C22—C23	173.1 (12)
C6—N1—C5—N2	0.5 (2)	C22—C21—C29—C30	61.5 (7)
C6—N1—C8—C9	−99.3 (2)	C22—C21—C29—C31	−62.9 (8)
C6—N1—C8—C13	76.6 (2)	C22—C21—C29A—C30A	87 (3)
C7—N2—C5—Cr	172.74 (14)	C22—C21—C29A—C31A	−37 (3)
C7—N2—C5—N1	−0.4 (2)	C22—C23—C24—C25	1.2 (3)
C7—N2—C20—C21	86.3 (2)	C23—C24—C25—C20	0.4 (3)
C7—N2—C20—C25	−90.2 (2)	C23—C24—C25—C26	−175.2 (7)
C8—N1—C5—Cr	7.3 (3)	C23—C24—C25—C26A	171.8 (10)
C8—N1—C5—N2	−179.56 (16)	C24—C25—C26—C27	−79.6 (12)
C8—N1—C6—C7	179.62 (19)	C24—C25—C26—C28	40.8 (15)
C8—C9—C17—C18	−143.1 (10)	C24—C25—C26A—C27A	−59.9 (18)
C8—C9—C17—C19	91.6 (11)	C24—C25—C26A—C28A	66.4 (19)
C8—C9—C17A—C18A	−130.6 (11)	C25—C20—C21—C29	−177.9 (3)
C8—C9—C17A—C19A	110.4 (15)	C25—C20—C21—C29A	−171.9 (11)
C8—C9—C10—C11	1.0 (3)	C25—C20—C21—C22	1.8 (3)
C8—C13—C14—C15	118.7 (2)	O5—C32—C33—C34	−43.2 (11)
C8—C13—C14—C16	−117.4 (2)	C32—O5—C35—C34	−9.6 (7)
C9—C8—C13—C12	3.4 (3)	C32—C33—C34—C35	36.1 (10)
C9—C8—C13—C14	−174.10 (19)	C33—C34—C35—O5	−17.4 (8)
C9—C10—C11—C12	1.6 (4)	C35—O5—C32—C33	33.3 (9)
C17—C9—C10—C11	−176.1 (6)	O5A—C32A—C33A—C34A	41 (4)
C17A—C9—C10—C11	176.7 (8)	C32A—O5A—C35A—C34A	−15 (3)
C10—C9—C17—C18	33.9 (13)	C32A—C33A—C34A—C35A	−50 (3)
C10—C9—C17—C19	−91.4 (12)	C33A—C34A—C35A—O5A	39 (2)
C10—C9—C17A—C18A	54.0 (17)	C35A—O5A—C32A—C33A	−16 (4)

Symmetry code: (i) −*x*+1, −*y*+1, −*z*+1.

### Tetrakis(µ-acetato-κ2O:O')bis{[1,3-bis(2,6-diisopropylphenyl)imidazol-2-ylidene-κC2]chromium(II)} tetrahydrofuran disolvate (1) Hydrogen-bond geometry (Å, º)

**Table:**

*D*—H···*A*	*D*—H	H···*A*	*D*···*A*	*D*—H···*A*
C6—H6···O5	0.95	2.47	3.411 (6)	171

### Tetrakis(µ-acetato-κ2O:O')bis{[1,3-bis(2,4,6-trimethylphenyl)imidazol-2-ylidene-κC2]chromium(II)}, (2) Crystal data

**Table:**

{Cr_2_(C_2_H_3_O_2_)_4_(C_21_H_24_N_2_)_2_]	*Z* = 1
*M**_r_* = 949.02	*F*(000) = 500
Triclinic, *P*1	*D*_x_ = 1.324 Mg m^−^^3^
*a* = 8.3679 (5) Å	Mo *K*α radiation, λ = 0.71073 Å
*b* = 11.6127 (8) Å	Cell parameters from 4925 reflections
*c* = 13.8355 (9) Å	θ = 2.0–29.2°
α = 68.949 (5)°	µ = 0.51 mm^−^^1^
β = 83.891 (5)°	*T* = 170 K
γ = 71.508 (5)°	Plate, clear violet
*V* = 1189.90 (14) Å^3^	0.40 × 0.24 × 0.07 mm

### Tetrakis(µ-acetato-κ2O:O')bis{[1,3-bis(2,4,6-trimethylphenyl)imidazol-2-ylidene-κC2]chromium(II)}, (2) Data collection

**Table:**

Stoe IPDS 2T diffractometer	4174 independent reflections
Radiation source: sealed X-ray tube, 12 x 0.4 mm long-fine focus	2894 reflections with *I* > 2σ(*I*)
Plane graphite monochromator	*R*_int_ = 0.071
Detector resolution: 6.67 pixels mm^-1^	θ_max_ = 25.0°, θ_min_ = 2.1°
rotation method, ω scans	*h* = −8→9
Absorption correction: numerical (X-RED32; Stoe & Cie, 2015)	*k* = −13→13
*T*_min_ = 0.844, *T*_max_ = 0.963	*l* = −16→16
8474 measured reflections	

### Tetrakis(µ-acetato-κ2O:O')bis{[1,3-bis(2,4,6-trimethylphenyl)imidazol-2-ylidene-κC2]chromium(II)}, (2) Refinement

**Table:**

Refinement on *F*^2^	Primary atom site location: dual
Least-squares matrix: full	Hydrogen site location: inferred from neighbouring sites
*R*[*F*^2^ > 2σ(*F*^2^)] = 0.042	H-atom parameters constrained
*wR*(*F*^2^) = 0.112	*w* = 1/[σ^2^(*F*_o_^2^) + (0.0639*P*)^2^] where *P* = (*F*_o_^2^ + 2*F*_c_^2^)/3
*S* = 0.91	(Δ/σ)_max_ = 0.001
4174 reflections	Δρ_max_ = 0.50 e Å^−^^3^
297 parameters	Δρ_min_ = −0.51 e Å^−^^3^
0 restraints	

### Tetrakis(µ-acetato-κ2O:O')bis{[1,3-bis(2,4,6-trimethylphenyl)imidazol-2-ylidene-κC2]chromium(II)}, (2) Special details

**Table:**

Geometry. All esds (except the esd in the dihedral angle between two l.s. planes) are estimated using the full covariance matrix. The cell esds are taken into account individually in the estimation of esds in distances, angles and torsion angles; correlations between esds in cell parameters are only used when they are defined by crystal symmetry. An approximate (isotropic) treatment of cell esds is used for estimating esds involving l.s. planes.

### Tetrakis(µ-acetato-κ2O:O')bis{[1,3-bis(2,4,6-trimethylphenyl)imidazol-2-ylidene-κC2]chromium(II)}, (2) Fractional atomic coordinates and isotropic or equivalent isotropic displacement parameters (Å^2^)

**Table:**

	*x*	*y*	*z*	*U*_iso_*/*U*_eq_	
Cr	0.54824 (5)	0.51154 (4)	0.40816 (3)	0.02630 (15)	
O1	0.3003 (2)	0.59224 (19)	0.36961 (14)	0.0351 (5)	
N1	0.5905 (3)	0.6422 (2)	0.14863 (17)	0.0343 (6)	
C1	0.1879 (3)	0.6037 (3)	0.4367 (2)	0.0316 (6)	
O2	0.2154 (2)	0.57212 (18)	0.53188 (15)	0.0340 (4)	
N2	0.7496 (3)	0.4481 (2)	0.19833 (17)	0.0330 (5)	
C2	0.0074 (3)	0.6573 (3)	0.4002 (3)	0.0445 (8)	
H2A	−0.061866	0.700386	0.446329	0.067*	
H2B	−0.033270	0.586646	0.400891	0.067*	
H2C	−0.000188	0.719885	0.329572	0.067*	
O3	0.5662 (2)	0.68514 (17)	0.39901 (14)	0.0338 (4)	
C3	0.5333 (3)	0.7243 (3)	0.4751 (2)	0.0309 (6)	
O4	0.4828 (2)	0.66423 (18)	0.56159 (14)	0.0335 (4)	
C4	0.5595 (4)	0.8516 (3)	0.4611 (3)	0.0454 (8)	
H4A	0.482967	0.891917	0.506614	0.068*	
H4B	0.536228	0.908816	0.388898	0.068*	
H4C	0.676311	0.837131	0.478780	0.068*	
C5	0.6360 (3)	0.5358 (3)	0.2356 (2)	0.0285 (6)	
C6	0.6703 (4)	0.6201 (3)	0.0613 (2)	0.0478 (8)	
H6	0.656021	0.679986	−0.007647	0.057*	
C7	0.7702 (4)	0.4995 (3)	0.0922 (2)	0.0474 (8)	
H7	0.842308	0.456314	0.049825	0.057*	
C8	0.4637 (3)	0.7630 (3)	0.1422 (2)	0.0314 (6)	
C9	0.5118 (3)	0.8645 (3)	0.1481 (2)	0.0327 (6)	
C10	0.3881 (4)	0.9817 (3)	0.1331 (2)	0.0371 (7)	
H10	0.418452	1.052446	0.136277	0.044*	
C11	0.2212 (4)	0.9993 (3)	0.1135 (2)	0.0370 (7)	
C12	0.1785 (4)	0.8955 (3)	0.1109 (2)	0.0382 (7)	
H12	0.064049	0.906087	0.099248	0.046*	
C13	0.2971 (3)	0.7752 (3)	0.1248 (2)	0.0354 (7)	
C14	0.6915 (4)	0.8479 (3)	0.1710 (2)	0.0441 (8)	
H14A	0.707584	0.820080	0.246043	0.066*	
H14B	0.715899	0.930470	0.137280	0.066*	
H14C	0.767964	0.782500	0.144690	0.066*	
C15	0.0910 (4)	1.1303 (3)	0.0923 (2)	0.0505 (8)	
H15A	0.110195	1.171200	0.138957	0.076*	
H15B	−0.021906	1.119692	0.103718	0.076*	
H15C	0.100211	1.184860	0.020298	0.076*	
C16	0.2480 (4)	0.6630 (3)	0.1227 (2)	0.0479 (8)	
H16A	0.272811	0.593565	0.190257	0.072*	
H16B	0.311952	0.631495	0.068962	0.072*	
H16C	0.127199	0.690516	0.107617	0.072*	
C17	0.8384 (3)	0.3160 (3)	0.2571 (2)	0.0317 (6)	
C18	0.7640 (3)	0.2203 (3)	0.2707 (2)	0.0348 (7)	
C19	0.8564 (4)	0.0938 (3)	0.3208 (2)	0.0391 (7)	
H19	0.806511	0.027229	0.331513	0.047*	
C20	1.0202 (4)	0.0600 (3)	0.3562 (2)	0.0387 (7)	
C21	1.0873 (3)	0.1596 (3)	0.3422 (2)	0.0374 (7)	
H21	1.198156	0.138463	0.367078	0.045*	
C22	1.0007 (3)	0.2876 (3)	0.2938 (2)	0.0334 (6)	
C23	0.5886 (4)	0.2541 (3)	0.2299 (3)	0.0494 (8)	
H23A	0.511481	0.320742	0.254719	0.074*	
H23B	0.550978	0.176680	0.254475	0.074*	
H23C	0.589497	0.286814	0.153996	0.074*	
C24	1.1213 (4)	−0.0789 (3)	0.4055 (3)	0.0538 (9)	
H24A	1.186112	−0.111074	0.352228	0.081*	
H24B	1.045268	−0.130492	0.439253	0.081*	
H24C	1.198730	−0.085845	0.457128	0.081*	
C25	1.0787 (4)	0.3930 (3)	0.2774 (3)	0.0509 (8)	
H25A	0.995447	0.465874	0.291890	0.076*	
H25B	1.115855	0.422015	0.205433	0.076*	
H25C	1.175804	0.359940	0.324063	0.076*	

### Tetrakis(µ-acetato-κ2O:O')bis{[1,3-bis(2,4,6-trimethylphenyl)imidazol-2-ylidene-κC2]chromium(II)}, (2) Atomic displacement parameters (Å^2^)

**Table:**

	*U*^11^	*U*^22^	*U*^33^	*U*^12^	*U*^13^	*U*^23^
Cr	0.0215 (2)	0.0263 (2)	0.0304 (3)	−0.00359 (17)	−0.00045 (17)	−0.01198 (19)
O1	0.0261 (10)	0.0418 (12)	0.0357 (11)	−0.0044 (9)	−0.0027 (8)	−0.0156 (9)
N1	0.0347 (13)	0.0312 (14)	0.0326 (13)	−0.0035 (10)	−0.0001 (10)	−0.0116 (11)
C1	0.0226 (14)	0.0258 (15)	0.0459 (18)	−0.0048 (11)	−0.0048 (13)	−0.0124 (13)
O2	0.0230 (9)	0.0395 (12)	0.0385 (12)	−0.0036 (8)	0.0003 (8)	−0.0173 (9)
N2	0.0336 (13)	0.0306 (13)	0.0316 (13)	−0.0026 (10)	0.0019 (10)	−0.0135 (11)
C2	0.0255 (14)	0.049 (2)	0.057 (2)	−0.0019 (13)	−0.0101 (14)	−0.0212 (16)
O3	0.0381 (11)	0.0285 (11)	0.0374 (11)	−0.0104 (9)	0.0028 (9)	−0.0149 (9)
C3	0.0202 (13)	0.0268 (15)	0.0461 (18)	−0.0042 (11)	−0.0021 (12)	−0.0150 (14)
O4	0.0372 (11)	0.0300 (11)	0.0368 (11)	−0.0108 (9)	0.0037 (9)	−0.0160 (9)
C4	0.0453 (18)	0.0352 (18)	0.062 (2)	−0.0136 (14)	0.0060 (15)	−0.0245 (16)
C5	0.0257 (13)	0.0298 (16)	0.0298 (15)	−0.0061 (12)	−0.0016 (11)	−0.0115 (12)
C6	0.061 (2)	0.047 (2)	0.0252 (16)	−0.0061 (17)	0.0065 (14)	−0.0101 (15)
C7	0.0556 (19)	0.044 (2)	0.0323 (17)	−0.0005 (16)	0.0110 (14)	−0.0175 (15)
C8	0.0355 (15)	0.0264 (15)	0.0260 (14)	−0.0044 (12)	−0.0025 (12)	−0.0053 (12)
C9	0.0337 (15)	0.0355 (17)	0.0251 (14)	−0.0095 (13)	0.0007 (12)	−0.0071 (12)
C10	0.0459 (17)	0.0294 (16)	0.0347 (16)	−0.0094 (13)	−0.0022 (13)	−0.0106 (13)
C11	0.0405 (17)	0.0349 (17)	0.0275 (15)	−0.0019 (13)	0.0014 (13)	−0.0097 (13)
C12	0.0304 (15)	0.0437 (19)	0.0332 (16)	−0.0049 (13)	−0.0049 (12)	−0.0089 (14)
C13	0.0371 (15)	0.0378 (17)	0.0269 (15)	−0.0094 (13)	−0.0049 (12)	−0.0065 (13)
C14	0.0355 (16)	0.051 (2)	0.0490 (19)	−0.0162 (15)	0.0007 (14)	−0.0178 (16)
C15	0.0502 (19)	0.043 (2)	0.0460 (19)	0.0061 (15)	−0.0021 (15)	−0.0173 (16)
C16	0.0486 (18)	0.045 (2)	0.053 (2)	−0.0170 (16)	−0.0117 (16)	−0.0142 (16)
C17	0.0313 (14)	0.0310 (16)	0.0325 (15)	−0.0045 (12)	0.0061 (12)	−0.0163 (13)
C18	0.0317 (15)	0.0391 (18)	0.0376 (16)	−0.0087 (13)	0.0040 (12)	−0.0207 (14)
C19	0.0404 (16)	0.0386 (18)	0.0460 (18)	−0.0155 (14)	0.0052 (14)	−0.0216 (15)
C20	0.0408 (16)	0.0324 (17)	0.0394 (17)	−0.0059 (13)	0.0003 (13)	−0.0125 (14)
C21	0.0284 (15)	0.0413 (18)	0.0440 (17)	−0.0042 (13)	−0.0017 (13)	−0.0214 (14)
C22	0.0305 (14)	0.0348 (17)	0.0395 (16)	−0.0083 (13)	0.0048 (12)	−0.0208 (13)
C23	0.0357 (16)	0.061 (2)	0.060 (2)	−0.0147 (16)	−0.0014 (15)	−0.0297 (18)
C24	0.055 (2)	0.0368 (19)	0.061 (2)	−0.0012 (16)	−0.0054 (17)	−0.0173 (17)
C25	0.0439 (18)	0.049 (2)	0.071 (2)	−0.0189 (16)	0.0035 (16)	−0.0300 (18)

### Tetrakis(µ-acetato-κ2O:O')bis{[1,3-bis(2,4,6-trimethylphenyl)imidazol-2-ylidene-κC2]chromium(II)}, (2) Geometric parameters (Å, º)

**Table:**

Cr—Cr^i^	2.5284 (9)	C11—C15	1.511 (4)
Cr—O1	2.0274 (18)	C12—H12	0.9500
Cr—O2^i^	2.0238 (17)	C12—C13	1.393 (4)
Cr—O3	2.0269 (18)	C13—C16	1.495 (4)
Cr—O4^i^	2.0270 (18)	C14—H14A	0.9800
Cr—C5	2.365 (3)	C14—H14B	0.9800
O1—C1	1.260 (3)	C14—H14C	0.9800
N1—C5	1.360 (3)	C15—H15A	0.9800
N1—C6	1.384 (4)	C15—H15B	0.9800
N1—C8	1.445 (3)	C15—H15C	0.9800
C1—O2	1.260 (3)	C16—H16A	0.9800
C1—C2	1.504 (3)	C16—H16B	0.9800
N2—C5	1.366 (3)	C16—H16C	0.9800
N2—C7	1.388 (4)	C17—C18	1.385 (4)
N2—C17	1.441 (3)	C17—C22	1.398 (4)
C2—H2A	0.9800	C18—C19	1.378 (4)
C2—H2B	0.9800	C18—C23	1.510 (4)
C2—H2C	0.9800	C19—H19	0.9500
O3—C3	1.263 (3)	C19—C20	1.392 (4)
C3—O4	1.255 (3)	C20—C21	1.384 (4)
C3—C4	1.504 (4)	C20—C24	1.500 (4)
C4—H4A	0.9800	C21—H21	0.9500
C4—H4B	0.9800	C21—C22	1.374 (4)
C4—H4C	0.9800	C22—C25	1.500 (4)
C6—H6	0.9500	C23—H23A	0.9800
C6—C7	1.321 (4)	C23—H23B	0.9800
C7—H7	0.9500	C23—H23C	0.9800
C8—C9	1.391 (4)	C24—H24A	0.9800
C8—C13	1.394 (4)	C24—H24B	0.9800
C9—C10	1.384 (4)	C24—H24C	0.9800
C9—C14	1.508 (4)	C25—H25A	0.9800
C10—H10	0.9500	C25—H25B	0.9800
C10—C11	1.387 (4)	C25—H25C	0.9800
C11—C12	1.375 (4)		
			
O1—Cr—Cr^i^	85.57 (6)	C12—C11—C10	118.3 (3)
O1—Cr—C5	93.88 (8)	C12—C11—C15	120.9 (3)
O2^i^—Cr—Cr^i^	86.09 (6)	C11—C12—H12	118.8
O2^i^—Cr—O1	171.65 (8)	C11—C12—C13	122.4 (3)
O2^i^—Cr—O3	88.82 (7)	C13—C12—H12	118.8
O2^i^—Cr—O4^i^	91.03 (7)	C8—C13—C16	121.2 (3)
O2^i^—Cr—C5	94.46 (8)	C12—C13—C8	117.2 (3)
O3—Cr—Cr^i^	85.62 (6)	C12—C13—C16	121.6 (3)
O3—Cr—O1	90.66 (8)	C9—C14—H14A	109.5
O3—Cr—C5	93.90 (8)	C9—C14—H14B	109.5
O4^i^—Cr—Cr^i^	86.03 (6)	C9—C14—H14C	109.5
O4^i^—Cr—O1	88.28 (7)	H14A—C14—H14B	109.5
O4^i^—Cr—O3	171.63 (8)	H14A—C14—H14C	109.5
O4^i^—Cr—C5	94.45 (8)	H14B—C14—H14C	109.5
C5—Cr—Cr^i^	179.26 (7)	C11—C15—H15A	109.5
C1—O1—Cr	121.90 (16)	C11—C15—H15B	109.5
C5—N1—C6	112.2 (2)	C11—C15—H15C	109.5
C5—N1—C8	125.9 (2)	H15A—C15—H15B	109.5
C6—N1—C8	121.7 (2)	H15A—C15—H15C	109.5
O1—C1—C2	117.3 (2)	H15B—C15—H15C	109.5
O2—C1—O1	124.9 (2)	C13—C16—H16A	109.5
O2—C1—C2	117.8 (2)	C13—C16—H16B	109.5
C1—O2—Cr^i^	121.50 (16)	C13—C16—H16C	109.5
C5—N2—C7	111.9 (2)	H16A—C16—H16B	109.5
C5—N2—C17	126.7 (2)	H16A—C16—H16C	109.5
C7—N2—C17	121.4 (2)	H16B—C16—H16C	109.5
C1—C2—H2A	109.5	C18—C17—N2	119.0 (2)
C1—C2—H2B	109.5	C18—C17—C22	122.1 (3)
C1—C2—H2C	109.5	C22—C17—N2	118.8 (2)
H2A—C2—H2B	109.5	C17—C18—C23	120.8 (3)
H2A—C2—H2C	109.5	C19—C18—C17	117.9 (2)
H2B—C2—H2C	109.5	C19—C18—C23	121.3 (3)
C3—O3—Cr	121.83 (18)	C18—C19—H19	118.8
O3—C3—C4	117.2 (3)	C18—C19—C20	122.3 (3)
O4—C3—O3	124.9 (2)	C20—C19—H19	118.8
O4—C3—C4	117.8 (2)	C19—C20—C24	121.3 (3)
C3—O4—Cr^i^	121.56 (16)	C21—C20—C19	117.4 (3)
C3—C4—H4A	109.5	C21—C20—C24	121.4 (3)
C3—C4—H4B	109.5	C20—C21—H21	118.5
C3—C4—H4C	109.5	C22—C21—C20	123.0 (2)
H4A—C4—H4B	109.5	C22—C21—H21	118.5
H4A—C4—H4C	109.5	C17—C22—C25	121.0 (3)
H4B—C4—H4C	109.5	C21—C22—C17	117.4 (3)
N1—C5—Cr	128.80 (18)	C21—C22—C25	121.6 (3)
N1—C5—N2	102.3 (2)	C18—C23—H23A	109.5
N2—C5—Cr	128.90 (19)	C18—C23—H23B	109.5
N1—C6—H6	126.6	C18—C23—H23C	109.5
C7—C6—N1	106.8 (3)	H23A—C23—H23B	109.5
C7—C6—H6	126.6	H23A—C23—H23C	109.5
N2—C7—H7	126.6	H23B—C23—H23C	109.5
C6—C7—N2	106.8 (3)	C20—C24—H24A	109.5
C6—C7—H7	126.6	C20—C24—H24B	109.5
C9—C8—N1	119.5 (2)	C20—C24—H24C	109.5
C9—C8—C13	122.3 (3)	H24A—C24—H24B	109.5
C13—C8—N1	118.0 (2)	H24A—C24—H24C	109.5
C8—C9—C14	121.6 (3)	H24B—C24—H24C	109.5
C10—C9—C8	117.6 (2)	C22—C25—H25A	109.5
C10—C9—C14	120.9 (3)	C22—C25—H25B	109.5
C9—C10—H10	118.9	C22—C25—H25C	109.5
C9—C10—C11	122.2 (3)	H25A—C25—H25B	109.5
C11—C10—H10	118.9	H25A—C25—H25C	109.5
C10—C11—C15	120.8 (3)	H25B—C25—H25C	109.5
			
Cr—O1—C1—O2	2.9 (4)	C8—N1—C5—Cr	−6.1 (4)
Cr—O1—C1—C2	−176.25 (18)	C8—N1—C5—N2	176.0 (2)
Cr—O3—C3—O4	−2.0 (3)	C8—N1—C6—C7	−176.2 (3)
Cr—O3—C3—C4	176.97 (17)	C8—C9—C10—C11	0.5 (4)
O1—C1—O2—Cr^i^	−3.0 (4)	C9—C8—C13—C12	1.4 (4)
N1—C6—C7—N2	0.6 (4)	C9—C8—C13—C16	−177.7 (3)
N1—C8—C9—C10	174.8 (2)	C9—C10—C11—C12	1.1 (4)
N1—C8—C9—C14	−5.8 (4)	C9—C10—C11—C15	−177.0 (3)
N1—C8—C13—C12	−175.2 (2)	C10—C11—C12—C13	−1.5 (4)
N1—C8—C13—C16	5.7 (4)	C11—C12—C13—C8	0.3 (4)
N2—C17—C18—C19	175.5 (2)	C11—C12—C13—C16	179.4 (3)
N2—C17—C18—C23	−3.4 (4)	C13—C8—C9—C10	−1.8 (4)
N2—C17—C22—C21	−174.8 (2)	C13—C8—C9—C14	177.7 (3)
N2—C17—C22—C25	3.1 (4)	C14—C9—C10—C11	−179.0 (3)
C2—C1—O2—Cr^i^	176.10 (18)	C15—C11—C12—C13	176.5 (3)
O3—C3—O4—Cr^i^	1.5 (3)	C17—N2—C5—Cr	2.8 (4)
C4—C3—O4—Cr^i^	−177.53 (17)	C17—N2—C5—N1	−179.3 (2)
C5—N1—C6—C7	−1.2 (4)	C17—N2—C7—C6	178.7 (3)
C5—N1—C8—C9	96.3 (3)	C17—C18—C19—C20	−0.9 (4)
C5—N1—C8—C13	−87.0 (3)	C18—C17—C22—C21	1.6 (4)
C5—N2—C7—C6	0.1 (4)	C18—C17—C22—C25	179.5 (3)
C5—N2—C17—C18	87.9 (3)	C18—C19—C20—C21	2.0 (4)
C5—N2—C17—C22	−95.5 (3)	C18—C19—C20—C24	−176.7 (3)
C6—N1—C5—Cr	179.1 (2)	C19—C20—C21—C22	−1.2 (4)
C6—N1—C5—N2	1.2 (3)	C20—C21—C22—C17	−0.5 (4)
C6—N1—C8—C9	−89.3 (3)	C20—C21—C22—C25	−178.4 (3)
C6—N1—C8—C13	87.3 (3)	C22—C17—C18—C19	−1.0 (4)
C7—N2—C5—Cr	−178.7 (2)	C22—C17—C18—C23	−179.9 (3)
C7—N2—C5—N1	−0.8 (3)	C23—C18—C19—C20	178.0 (3)
C7—N2—C17—C18	−90.5 (3)	C24—C20—C21—C22	177.4 (3)
C7—N2—C17—C22	86.1 (3)		

Symmetry code: (i) −*x*+1, −*y*+1, −*z*+1.

### Tetrakis(µ-acetato-κ2O:O')bis{[1,3-bis(2,4,6-trimethylphenyl)imidazol-2-ylidene-κC2]chromium(II)}, (2) Hydrogen-bond geometry (Å, º)

*Cg* is the centroid of the C17–C22 ring.

**Table:**

*D*—H···*A*	*D*—H	H···*A*	*D*···*A*	*D*—H···*A*
C2—H2*B*···O2^ii^	0.98	2.61	3.527 (4)	155
C15—H15*A*···*Cg*^iii^	0.98	2.62	3.340 (3)	125

Symmetry codes: (ii) −*x*, −*y*+1, −*z*+1; (iii) *x*−1, *y*+1, *z*.
